# Uniparental analysis of Deep Maniot Greeks reveals genetic continuity from the pre-Medieval era

**DOI:** 10.1038/s42003-026-09597-9

**Published:** 2026-02-04

**Authors:** Leonidas-Romanos Davranoglou, Athanasios Petros Kofinakos, Anargyros D. Mariolis, Göran Runfeldt, Paul Andrew Maier, Michael Sager, Panagiota Soulioti, Theodoros Mariolis-Sapsakos, Alexandros Heraclides

**Affiliations:** 1 The Steinhardt Museum, Tel Aviv University, Tel Aviv, Israel; 2 Oxford University Museum of Natural History, University of Oxford, Oxford, UK; 3 Faculty of Nursing, National and Kapodistrian University of Athens, Athens, Greece; 4 Independent Researcher, Piraeus, Greece; 5 Areopolis Health Center, Areopolis, Greece; 6 FamilyTreeDNA, Gene by Gene, Houston, TX USA; 7 School of Sciences, European University Cyprus, Nicosia, Cyprus

**Keywords:** Population genetics, History

## Abstract

The Deep Maniots, an isolated population at the southernmost tip of mainland Greece, have drawn scholarly interest for their unique dialect, culture, and patrilineal clan structure. Geographically shielded by the Mani Peninsula, they are thought to have been minimally affected by 6^th^-century CE migrations that transformed Balkan demography. To investigate their genetic origins, we analysed Y-DNA and mtDNA from 102 Deep Maniots using next-generation sequencing. Paternally, Deep Maniots exhibit an exceptional prevalence (~80%) of West Asian haplogroup J-M172 (J2a), with subclade J-L930 accounting for ~50% of lineages. We identify Bronze Age Greek ancestry in Y-haplogroups nearly absent elsewhere, highlighting their longstanding genetic isolation. The absence of northeast European-related paternal lineages, common in other mainland Greeks, suggests preservation of southern Greece’s pre-Medieval genetic landscape. Y-haplogroup phylogeny reveals strong founder effects dated to ~380–670 CE, while the emergence of clan-based social structure is estimated around 1350 CE, centuries earlier than previously thought. In contrast, maternal lineages display greater heterogeneity, primarily originating from ancient Balkan, Levantine, and West Eurasian sources. These results align with historical and anthropological accounts, showcasing Deep Maniots as a genetic snapshot of pre-Medieval southern Greece, offering new perspectives on population continuity and mobility in the Late Antique eastern Mediterranean.

## Abstract (web summary)

Deep Maniot Greeks preserve Y-DNA and mtDNA from Bronze-to-Roman Age Greece, largely untouched by later migrations. Genetics reveal historical demography, settlement patterns in southern Greece, and the emergence of their unique clan system

## Introduction

At the dawn of Late Antiquity (250 CE), the multiethnic Roman Empire reached its maximal geographical extent, spanning the entire Mediterranean, the Balkans, Asia Minor, parts of the Levant, and most of Western Europe^1^. Over the next centuries, during the Migration Period (ca. 300–700 CE), sociopolitical factors, epidemics, and the large-scale settlement of new peoples led to the widespread collapse of urban life across the Empire, severely affecting the inhabitants of the Balkan peninsula, including Greece^2,3^. The invasions and settlements of Slavic peoples into the territory of present-day Greece, which started in the 6^th^ century CE, led to social and demographic upheaval, as far south as the Peloponnese^4–7^. This period witnessed a reduction in the Greek-speaking population, a significant proportion of which sought refuge in fortified settlements and inaccessible mountainous regions^8^, while others learned to coexist with the Slavic newcomers^9^. Subsequent migrations and settlements by Crusader colonial states, as well as Albanian-speaking groups during the Late Medieval and Early Post-Medieval periods, further complicated the demographic and linguistic landscape of the Peloponnese^10^.

**Fig. 1:**
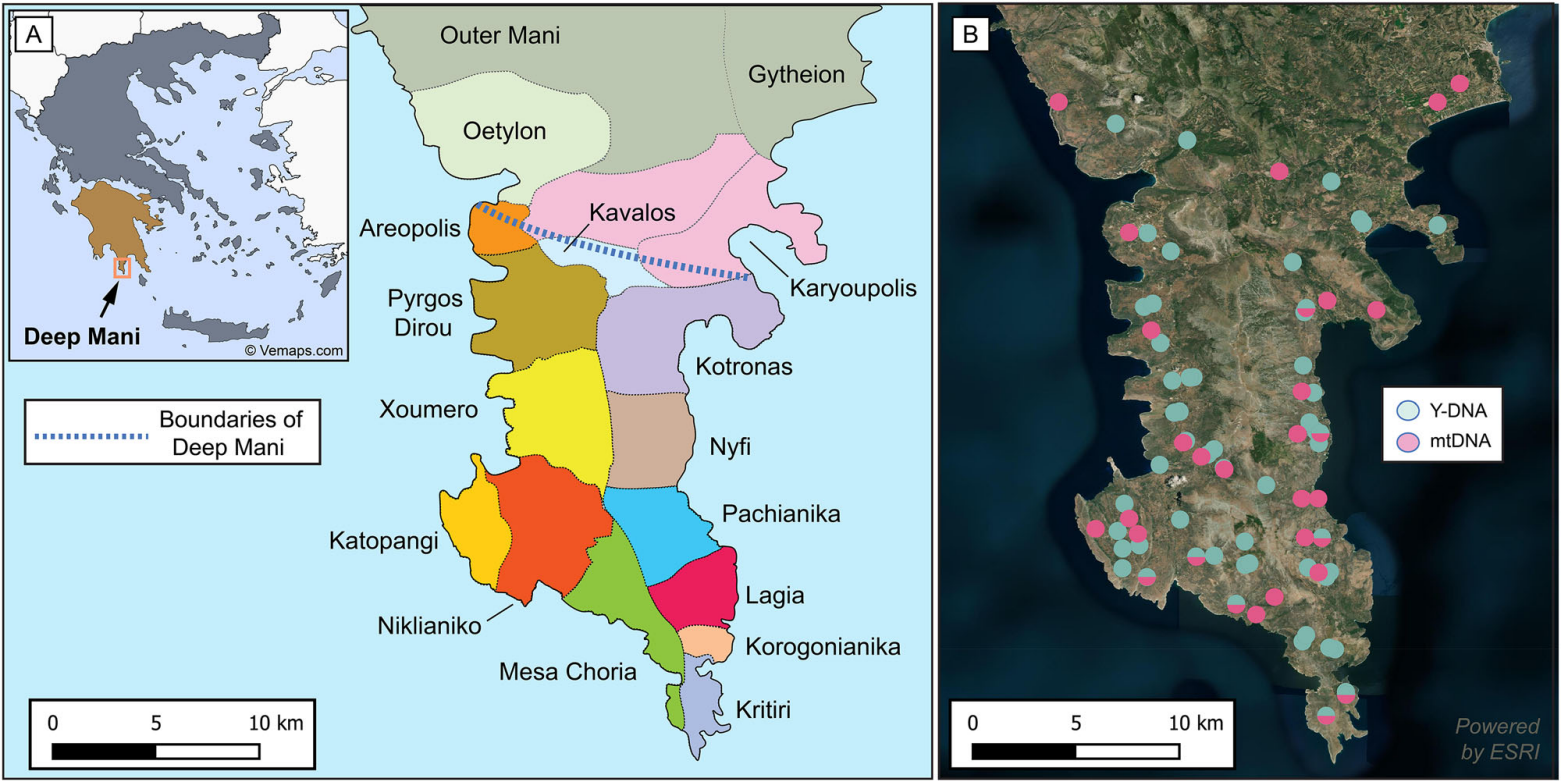
Outline of the Mani Peninsula. **A** Summary of traditional subdivisions of Deep Mani and adjacent areas. The blue dashed line indicates the approximate northern boundary of Deep Mani, according to linguistics, social organisation, and the emic perceptions of the region’s inhabitants^11,12,15^. Significant migrations from Deep Mani to Outer Mani and Gytheion after the 17^th^ century transformed the demography of those regions^12^. Inset map showing an outline of Greece, with Peloponnese highlighted in brown; arrow indicates the location of Deep Mani (inside red square). **B** Ancestral areas of origin of the newly sequenced present-day Deep Maniots. Each circle corresponds to the origin of each participant. The number of sampling sites (77) does not correspond to the total number of samples (102), as multiple individuals were sequenced from the same or adjacent locations. The satellite image of the Mani Peninsula was created with QGIS 3.40.0.48^155^ using basemaps from the Esri World Terrain Base Map, ArcGIS Online (Sources: Esri, USGS, NOAA)^156,157^, while the inset map of Greece is adapted from © Vemaps.com. (accessed 2025; https://vemaps.com/greece/gr-05).

The inhabitants of the Deep (or Mesa/Inner) Mani Peninsula, the Deep Maniots, historically resided in the southernmost tip of mainland Greece, in a region south of a virtual horizontal borderline starting from Areopolis in the west and finishing at Skoutari in the east^11,12^ (Fig. 1A). Historical, linguistic, and archaeological evidence suggests that Deep Maniots were minimally affected by the demographic turmoil of the Migration Period, most likely by blocking the southward advance of Slavic tribes who had settled extensively in the remainder of the Peloponnese^5,6,12–14^. Due to their geographic and cultural isolation in the Late Medieval and subsequent periods, Deep Maniots developed a unique local identity that differentiated them from other Greek speakers in the region^11,15^.

Due to the absence of a centralised administration and codified judicial system in their region during the Medieval era, the Deep Maniots developed a unique system of customary law that governed every aspect of social life^11,13^. A characteristic Maniot tradition was a complex, patrilineal clan system, which persisted until the mid-20^th^ century^5,11^. As a result, Deep Maniot society was highly stratified, characterised by patriarchal clans at the top of the social hierarchy, each claiming descent from a quasi-mythical, often noble, male ancestor who originated outside Mani, typically Constantinople, Asia Minor, Eastern Thrace, other regions of the Peloponnese, or Italy^11,15,16^. Clans occupied discrete, compartmentalised territories^11,13,15,16^ and at times engaged in inter-clan warfare and blood feuds, with members protecting themselves by constructing and residing in fortified tower houses that dot Mani’s landscape^13,15,16^. Weaker clans often lived within the territories of powerful clans, making alliances related to economic activities and warfare, while clan-less families occupied the lowest social tier and led a more vulnerable existence^11^.

Clans over time further branched out into subclans, with each subclan often comprising numerous distinct lineages and family names^11,15^. Most of the Deep Maniot clans surviving today are thought to have originated in the 16th–17^th^ centuries, based on the earliest census of Mani^11,12^. Whether the Deep Maniot clan system existed prior to this period remains an open question.

From the 18^th^ century onward, probably sparked by Western influences on Greek identity supporting direct continuity between the ancient and the modern Greek world, but also due to their militarised culture and the region’s historical ties to Sparta, many Deep Maniots increasingly associated themselves with the ancient Spartans^17^. Despite the plethora of perceived and suggested ancestries, there is no official consensus on the origins of Deep Maniots, puzzling chroniclers even 1100 years ago^18^. This uncertainty becomes clearer when examining the region’s turbulent history.

In classical antiquity, Mani was inhabited by poorly known, likely Doric-speaking tribes linked to Sparta and the broader Laconian world^19,20^. During Roman rule, Mani retained autonomy within the League of the Free Laconians (195 BCE–297 CE)^5^. After this period, there is practically no historical mention of Mani’s population apart from a successful defence against a Vandal invasion in the 5^th^ century CE^5,14,21^. Following these events, the fate of Deep Mani’s inhabitants remains obscure for more than 400 years. Whether present-day Deep Maniots descend from earlier local populations in the Mani Peninsula, resettled Greek-speakers, foreign settlers, or a mix of these, is unknown.

Archaeological and linguistic evidence suggests Deep Mani experienced a unique state of isolation compared to other rural Greek regions during this transitional era (5^th^–9^th^ centuries CE)^5,20^. This led to the abandonment of classical and Roman architectural styles, replaced by a unique megalithic tradition found exclusively in Deep Mani, preliminarily dated to the pre-Christian and early Christian periods^22,23^. Remarkably, the interior of Deep Mani may have remained pagan and without a monetary economy until the 9^th^ century CE^14,24^, despite the introduction of Christianity to coastal Mani during the 5^th^–8^th^ centuries CE^25^ and its expansion after 900 CE^26–29^. Evidently, Deep Maniots lived in a semi-autonomous enclave with distinct sociocultural traits, paying tribute instead of taxes to the Eastern Roman Empire—a financial arrangement reserved for semi-integrated tribal confederations^30^. The first mention of the existence of Deep Maniots as a distinct population is historically documented by Eastern Roman Emperor Porphyrogenitus, who noted in the 10^th^ century CE that they ‘*are not of the lineage of the* [abovementioned] *Slavs, but of the Romans of old, who even today are called Hellenes by the locals on account of their former idolatry*’^18^. Over the following centuries, the long-standing isolation of Deep Maniots appears to have persisted and based on historical population estimates, they reached a total population of about 6000 individuals during the 15^th^ century^12^.

Due to the paucity of archaeological data and historical sources for the Early Medieval period, population genetics provide a unique opportunity to elucidate the origins of the Deep Maniots. Previously, an autosomal genetic analysis on Peloponnesian sub-populations, including a small number of Deep Maniots, found that the latter display very low levels of shared ancestry with modern Slavs from northeastern Europe^31^, which a subsequent study estimated to an average of 30% in other Peloponnesians^3,31^. Another study examined deep ancestral components (Neolithic, Mesolithic) of Deep Maniots, and their potential shared ancestry with southern Italian populations^32^. Despite these prior investigations, the actual ancestral origins of Deep Maniots and whether they were affected by demographic events in the region of present-day Greece have remained unstudied.

Extensive ancient DNA (aDNA) sampling has elucidated the genetic origins of Bronze and Iron Age populations of central and southern Greece^33–36^, although the ancestry of Greece’s Roman and Medieval population is largely unknown. Despite this, there is robust evidence that, during the Roman period, the Balkans received a large-scale influx of populations from Asia Minor and the Near East^3,37–39^. Based on their unique historical circumstances and linguistic particularities^40–45^, the Deep Maniots may represent a genetic snapshot of the pre-Migration Period Greek world, providing invaluable insights into human mobility of the post-classical Eastern Mediterranean.

Uniparental markers on the Y-chromosome and mitochondrial DNA (mtDNA) help trace human migrations and link present-day humans to ancient populations due to their unique inheritance patterns. These markers reveal demographic changes, migration and its gender dynamics, such as exogamy and sex bias^46–53^. They also help estimate historical population size, infer the emergence of new peoples and languages^33,48,54^, and test the reliability of historical records and oral traditions of shared genealogical descent^55–58^. Uniparental markers have never been explored among Deep Maniots, leading to a substantial knowledge gap.

To elucidate unknown aspects of Deep Maniot history, we studied 102 individuals with confirmed Deep Maniot ancestry on their paternal side, representing all major clans and families. Employing a state-of-the-art targeted enrichment protocol that uses 155,000 probes to sequence over 15 Mbp of the Y chromosome at high depth (35–105×), enabling full haplogroup resolution through analysis of 700 STRs and 750,000 SNPs^59,60^, we performed deep sequencing of the Y-chromosome on 71 Deep Maniots, supplementing with Y-STR (*n* = 14) and SNP-testing (*n* = 17) on the remaining 30 participants (Fig. 1B). mtDNA sequence data were also retrieved from 50 of the participants with maternal Deep Maniot ancestry. With this systematic approach, we aimed to uncover the paternal and maternal origins of the Deep Maniots, determine when the founding Deep Maniot population first appeared, and understand how it evolved over time and geographical space. Additionally, we investigate whether the Deep Maniot clan structure affected the distribution of paternal lineages and test the veracity of origin myths and kinship relationships of major clans. Overall, our study provides a comprehensive understanding of the genetic history and social structure of one of the most enigmatic Greek-speaking populations.

## Results

### Deep Maniots represent an isolated genetic island within mainland Greece

**Fig. 2:**
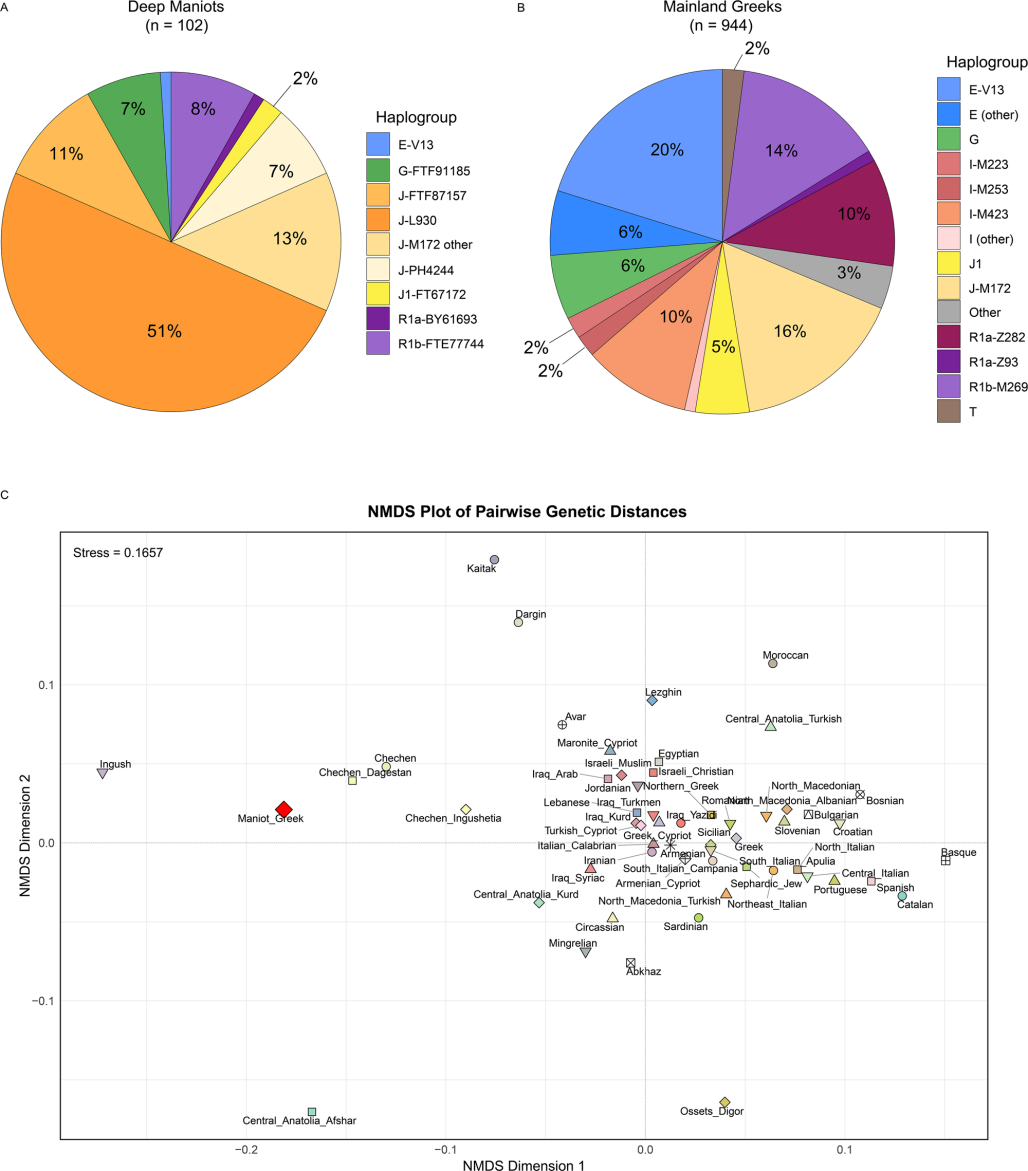
Comparative distribution of major Y-DNA haplogroups in **A** Deep Maniots and **B** other mainland Greeks. **C** NMDS plot derived from Rogers’ pairwise distances of 17 Y-STRs of 94 west Eurasian populations. The NMDS plot visualises Dimension 1 versus Dimension 2. The list of populations can be found in Supplementary Data 5.

Our analysis of 102 Deep Maniots revealed the presence of 14 distinct Y-chromosome haplogroups, which are summarised in Supplementary Data 1. Deep Maniots are characterised by an extremely high frequency of macro-haplogroup J-M172 (J2a) at over 80% (Fig. 2A), second only to the Ingush (88%)^61^, an ethnic group from the Caucasus. Remarkably, the frequency of this haplogroup in mainland Greece does not exceed 16% (Supplementary Data 2), peaking in the island of Crete at 30–35%^62,63^. In the broader Eastern Mediterranean region, the highest frequencies of J-M172 can be found among Greek Cypriots and the Lebanese, at ∼30%^63–65^, much lower than what observed among Deep Maniots.

The J-M172 lineage traces its origins to the Caucasus-Zagros region ca. 28,000–26,000 BCE, according to the latest FamilyTreeDNA^66^ and Yfull^67^ estimates, and its earliest aDNA record is in Mesolithic Caucasus Hunter Gatherers^68^ and Neolithic farmers from present-day Iran^69^, during the 8^th^ millennium BCE. Nine of the 14 haplogroups found in Deep Maniots belong to J-M172 (Supplementary Data 1), and the remaining lineages are assigned to macro-haplogroups R1b-Z2103 (R1b1a1b1b; 8%), G-L13 (G2a2b2a1a1a1a; 7%), J1 (2%), E-V13 (E1b1b1a1b1a; 1%) and R1a-Z93 (R1a1a1b2; 1%) (Fig. 2A). The phylogeographic history of each Deep Maniot lineage is analysed extensively in subsequent sections of this study.

As noted above, Deep Maniot Y-chromosome haplogroup distributions are markedly different compared to a sample of 944 citizens of mainland Greece sourced from the literature (Fig. 2A, B). Lineages associated with Germanic (I-M253) and Slavic (I-CTS10228, R1a-Z282) peoples^3,70^, who massively settled the Balkans during the Migration Period^3^, are found in a combined frequency of 22% in present-day mainland Greeks (Fig. 2B, Supplementary Data 2). Notably, these lineages are entirely absent from the Deep Maniot dataset (Fig. 2A, Supplementary Data 1, 2), suggesting limited to no paternal contribution from Germanic and Slavic peoples. The low incidence of Y-haplogroup J1 (J-M267) (2%) in Deep Mani (Fig. 2A) also indicates limited contribution from Levantine populations, where this haplogroup is found today in significant frequencies (23–66%)^49,66^ (some J1 lineages are also known from BA Greece)^35^. The Deep Maniot sample also lacks Y-haplogroups associated with the Central-Northern European (R1b-U152, R1b-L2, R1b-U106)^71,72^ and Albanian expansions (e.g. R1b-BY611)^73^ into Greece in the Middle Ages. Remarkably, Y-haplogroup E-V13, the dominant paternal lineage of present-day mainland Greeks (20%; Fig. 2B), is found in very low frequencies in Deep Mani (1%). Although E-V13 was previously thought to have been transmitted by archaic Greeks^74^, it is so far entirely absent in ancient samples from Bronze and Iron Age central and southern Greece (Supplementary Data 3), with its earliest record in the Hellenistic era^75^. A recent study has suggested the large-scale dissemination of this haplogroup occurred in at least three major pulses: one in the Iron Age and Roman Period with populations located north of present-day Greece, such as the ancient Daco-Thracians, one with Aromanians and another with Albanians in the Middle Ages^73^. The disparities in the frequencies of major haplogroups between mainland Greeks and Deep Maniots demonstrate that the latter were minimally affected by demographic events that shaped the paternal genetic landscape of Balkan populations during the Migration and Medieval Periods.

To visualise the paternal genetic relationships of Deep Maniots (17 STRs available for 75 individuals) with a large dataset (*n* = 12,647) of 94 west Eurasian populations grouped into 61 metapopulations, including 405 mainland Greeks (Supplementary Data 4), we applied Non-Metric Multidimensional Scaling (NMDS) on pairwise Rogers distances^76^ calculated on 17 Y-chromosome STRs (Fig. 2C; Supplementary Data 5). The NMDS plot effectively captures the expected relationships of the populations in the dataset (Fig. 2C) based on geography, previously demonstrated genetic relatedness, and the low stress value of 0.1657. Notable examples include Greek and Turkish Cypriots, who form a cohesive group, as expected^64,65^, while speakers of South Slavic languages cluster together compared to mainland Greeks and North Macedonian Albanians. Notably, Deep Maniots do not cluster close to any Balkan population, instead plotting towards Caucasian groups, especially with the outlying Ingush (Fig. 2C). This clustering pattern is evidently the result of the extremely high frequencies of haplogroup J-M172, shared by both populations, and does not reflect actual recent ancestry. Indeed, the J-M172 subclades found in Deep Maniots are entirely different to those of the Ingush, with the only related lineage, J-FTF77337, sharing a common ancestor with Caucasian populations at the root of J-Z1847 (J2a1a1a2b2a), at ca. 10,200 BCE^66^. We should also note that Y-chromosome haplogroups represent a single, non-recombinant marker, and therefore capture only a fraction of an individual’s ancestry. However, the paternal haplogroups of Deep Maniots clearly indicate that they are a genetic isolate within Greece, in accordance with previous studies based on autosomal ancestry^31^, indicating congruence between the two data sources.

To quantify genetic diversity within the Deep Maniot population, we estimated Nei’s haplotype diversity (*H*) at two levels of Y-STR resolution: using 111-locus haplotypes (*n* = 69) and 17-locus haplotypes (*n* = 75). We found that the 69 Y-111 STR haplotypes are unique (*H* = 1, i.e. there are no shared haplotypes between any of the Deep Maniot study participants), indicating population divergence over many centuries. Among the 75 Y-17 STR haplotypes, there are 44 unique haplotypes, of which 35 occur only once in the sample (Supplementary Data 6). The lower haplotype diversity at Y-17 (*H* ≈ 0.97) versus the maximal diversity at Y-111 (*H* = 1.00) primarily reflects the gain in discriminatory power when moving from 17 to 111 loci. When using only 17 Y-STR loci, the resolution is lower, meaning that distinct male lineages may appear identical due to limited marker variation. This results in a higher number of shared haplotypes within the Deep Maniot population, even among individuals with deep ancestral divergence.

Among the 44 unique Deep Maniot haplotypes at the Y-17 STR level, we did not find a single haplotype that was shared with individuals from other populations in our comparative dataset of nearly 13,000 west Eurasians, also including 405 mainland Greeks (Supplementary Data 4). When relaxing the matching criteria and allowing for ‘−1 matches’, only 11 matches were found for 5 Deep Maniot haplotypes (Supplementary Data 6). However, these matches reflect deep divergences (ca. 12,000–1,450 BCE; Supplementary Data 6) and are therefore not closely related to any Deep Maniot, except for a single Greek individual, who might belong to Deep Maniot-specific haplogroup J-L930 (see below). Our discovery that no exact haplotype is shared within our dataset of nearly 13,000 individuals from 94 population groups of interest, highlights the remarkable rarity of Deep Maniot haplotypes outside the Mani peninsula.

**Fig. 3:**
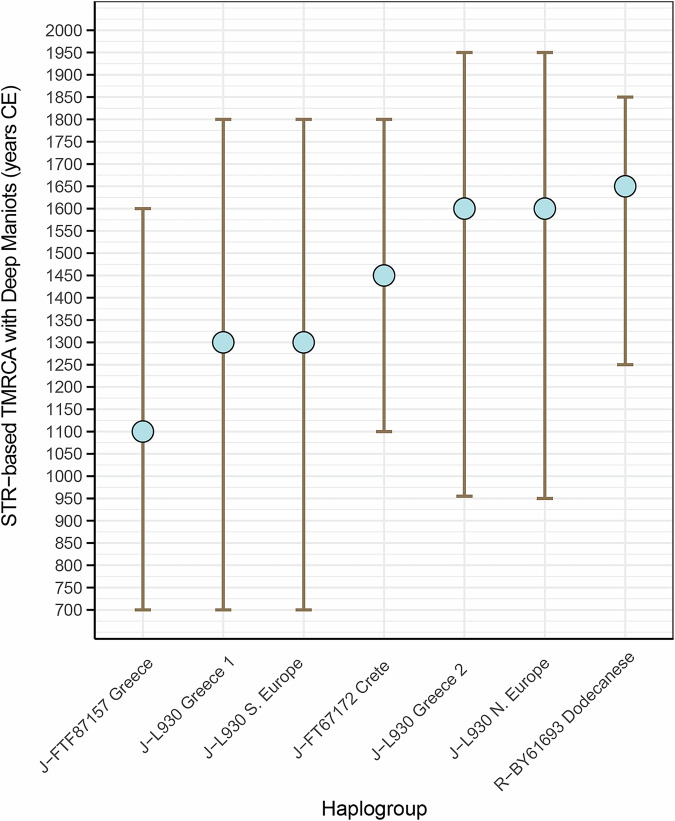
TMRCA estimation of the genetic affinities between non-Maniot individuals in the FamilyTreeDNA customer database who belong to Deep Maniot-specific Y-DNA haplogroups, based on 37–111 Y-STR markers. TMRCA estimates were derived using the FTDNATiP™ algorithm, which calculates coalescence dates from Y-STR genetic distances. Accuracy varies depending on the number of STR markers used, with higher-resolution profiles (e.g. 67–111 STRs) yielding narrower and more precise intervals than lower-resolution sets (e.g. 37 STRs). STR resolution for each sample is listed in Supplementary Data 6.

To increase the comparative strength of our search, we queried FamilyTreeDNA’s global customer Y-STR and Y-SNP database (>995,000 individuals). We found only 7 high-level STR matches (≥37 STRs) with non-Deep Maniots in the entire database (Supplementary Data 6). Using FamilyTreeDNA’s STR-based TMRCA estimation algorithm (FTDNATiP), we find that all these matches are recent—within the last 350–900 years, and 5/7 belong to Deep Maniot-specific lineages (J-L930, J-FTF87157, Fig. 3), probably representing descendants of Deep Maniots immigrating to other areas rather than migrations of outsiders into Deep Mani. We next queried FamilyTreeDNA’s >145,000 present-day user records at high sequence resolution^59^. Once more, we found only two Deep Maniot Y-haplogroups forming a subclade with non-Deep Maniots, at 750 BCE and 282 CE, respectively (Supplementary Data 6).

Overall, the STR and SNP-based comparative analyses and the outlying position of Deep Maniots on the NMDS plot (Fig. 2C) are consistent with patterns observed in genetically isolated and/or drifted groups, a term referring to populations that experienced random fluctuations in haplotype frequencies over successive generations due to chance variation in reproductive success^77,78^. The extreme frequency of J-M172 and the absence of shared haplotypes with other populations suggest that genetic drift, likely driven by long-term isolation and small effective population size, has potentially played an important role in shaping the Deep Maniot Y-chromosome landscape. Consequently, Deep Maniots represent a genetic island that has experienced longstanding isolation and has more likely acted as an exporter of genetic diversity to other parts of Greece and beyond, rather than a sink for newcomers. These results are congruent with historical sources that have extensively documented significant migrations out of Deep Mani towards Italy, Corsica, Balearic Islands, Asia Minor, and the Greek-speaking world more broadly (other parts of the Peloponnese, Ionian islands, Crete, Northern Aegean, Dodecanese) from the 16^th^ century onwards^12^. Although substantial relative to Deep Mani’s small population, these migrations may have left a limited demographic imprint on the regions they reached (with the exception of adjacent areas such as Gytheion), as suggested by the low number of genetic matches to non-Deep Maniots.

### Deep Maniots paternally descend from the Bronze Age and pre-Migration Period inhabitants of Greece

This section presents detailed phylogeographic information on the three most frequent Deep Maniot lineages (J-L930, J-FTF87157 and R-FTE77744), and their possible connections to populations from the past, as determined by aDNA. Each haplogroup is presented in an order that follows the presence of the earliest available related lineages in the aDNA record. A summary of all haplogroups and their phylogeographic associations are provided in Table 1, whereas for an extended discussion on the origins of the less frequent Deep Maniot Y-DNA lineages, refer to the S1 Text.

**Table 1:** Deep Maniot haplogroups and directly ancestral or related lineages in the aDNA record

Haplogroup (full path)	Closest ISOGG haplogroup	Frequency in Deep Mani	aDNA sample	Present-day distribution (FamilyTreeDNA/Yfull database)
J-M304>M172>M410>CTS7683>L26>PF5088>PF5160>PF5197>PF5172>Z7314>PF5169>PF5174>Z8074>PF7419>Z8072>PF7421>PF7423>FGC68843>Y31951>**L930**	J2a1a1b1a1a	51%	upstream branch J-Z8072 found in BA Caucasus (I2051)	J-L930 exclusively in Deep Mani; more distant branches (TMRCA in 2700 BCE) found in Greece, Cyprus, Albania, Levant, Iran, UK, Poland
J-M304>M172>M410>CTS7683>L26>Z6064>Y6858>Z6061>Z6057>Y7011>SK1359>PF7416>SK1363>BY759>PF7413>Z28527>Z35779>PF7415>FT88552>FTA97715>**FTF87157**	J2a1a2b2a2b2	11%	upstream branch J-Z35779 (1600 BCE) in Iron Age samples from Sicily with Aegean ancestry, Roman Period Rome; J-BY759 branches common in BA Greece	J-FTF87157 exclusively in Deep Mani; other branches in Poland, Italy, Iberia, Germany, Tunisia
R-M269>L23>Z2103>M12149>Z2106>FTE77744>**FTE77876**	R1b1a1b1b3	8%	early diverging R1b-Z2106 daughter lineages found in the Pontic-Caspian steppe, the Caucasus, and the Balkans	R-FTE77876 exclusively in Deep Mani. Sicilian match at 810 BCE
J-M304>M172>M410>CTS7683>L26>PF5088>PF5160>L24>Z393>L25>Z438>CTS1192>FGC35503>FGC35461>Z39097>**PH4244**	J2a1a1b2a1b2b	7%	Related branch J-Z40002 (2500 BCE) found in Byzantine Muğla (I20187)	Exclusively in Deep Mani. Sister branch J-Z39097>J-Z39121 (628 CE) found in Poland. Branches under J-FGC35461 largely West Asian
G-M201>L89>L156>P15>L1259>L30>L141>P303>L140>PF3346>Z3065>PF3345>Z6779>U1>FGC31390>L13>Z2024>Z2022>Z6414>PF6860>Z6759>FGC995>Z6764>FT1788 >FGC1016>S9751>Z29424>FGC31400>FT324622>FT275186>**FTF91185**	G2a2b2a1a1a1a1a1a1a	7%	Upstream branch G-FT324622 (1374 BCE) found in Roman Period individual (I26753; 200-300 CE) from Osijek, Croatia.	G-FTF91185 exclusive to Deep Mani. Sister branch G-FT275186>G-FT171308 (560 BCE) in Iran, Kuwait and Saudi Arabia
J-M304>M172>M410>CTS7683>L26>PF5088>PF5160>L24>Z393>L25>Z438>CTS1192>FGC35503>Z40002>Z39993>Z39995>Z40011>Z39986>Z40003>FT106400*	J2a1a1b2a1b2a	2%	Upstream branch J-Z40003 (930 BCE) found in Medieval Cambridgeshire (60; 1204-1511 CE). Related branch J-Z40002 (TMRCA at 2150 BCE) in Byzantine sample from Muğla (20259; 491-717 CE)	J-FT106400 found in Deep Mani. Downstream branches in Kuwait
J-M304>M172>M410>PF5050>PF5007>PF5058>Z39726>BY40937>Z28344>Z28333>FT151401>Z39727>FT151551*	J2a2a	2%	Same branch has been found in individual R2041 from Roman Period Sisak Pogorelec, Croatia.	No matches. Downstream branches in Czechia, Poland, Italy. Upstream branch J-Z39727 (1489 BCE) found in present-day Albanians, Cretans, Turks, Italians, and Germans
J-M304>M267>CTS12238>Z2217>L620>PF4832>L136>PF7264>ZS4381>PF7263>ZS4452>BY38106>BY45309>BY38105>BY137125>**FT67172**	J1a2a1a1b	2%	Ancient individual (I8216) from the classical Greek colony of Empuries in Spain was found to have upstream lineage J-PF7263.	J-FT67172 is found exclusively in Deep Mani and in Sicily. Related lineages in Italy, the Levant, Iran, Iberia, Greece, Central Asia, and northwestern Europe
J-M304>M172>M410>CTS7683>L26>PF5088>PF5160>L24>Z393>L25>Z438>CTS1192>L70>PF5430>Z435>Z2177>PH185>FGC24630>BY76232*	J2a1a1b2a1b1b2c	2%	None	Downstream branches found almost exclusively in Italy, with one lineage in the UK. Upstream lineage J-FGC24630 contains many branches in Anatolia, the Levant, Greece, Italy, and northwestern Europe.
J-M304>M172>M102>Z529>Z1827>Z593>M241>L283>Z622>Z600>Z2509>Z585>Z615>Z8418>Z597>Z2507>Z1296>Z1297>Z1295>Z8421>Z631*	J2b2a1a1a1a1a1a	2%	Both upstream and downstream branches largely in BA-IA, Roman, and Medieval Balkans	No matches
J-M304>M172>M410>CTS7683>L26>PF5088>PF5125>Z2227>Z1846>M67>Z1847>Y4036>Z467>Z455>L210>Z459>L227>FT114604>**Z44002**	J2a1a1a2b2a2b3a	1%	None	Matches a Swiss individual from 750 BCE. Downstream branches found in individuals in Central and Western Europe, as well as Iberia. Upstream branches in Ashkenazi Jews, the Balkans, Italy, Iran, and Western Europe.
J-M304>M172>M410>CTS7683>L26>PF5088>PF5160>L24>Z393>Z44288>Z44310>BY93436*	J2a1a1b2a	1%	None	No matches. Related branches in Armenia, Sudan, and the Arabian Peninsula
E-M96>CTS9083>P147>P177>P2>M215>M35>V68>M78>PF2179>Z1919>L618>CTS1975>V13>CTS8814>CTS5856>BY4877>BY3880>Y16729>BY202063>FT64983*	E1b1b1a1b1a14	1%	This lineage was found in a Roman Period individual from Trogir, Croatia (I26702)	No matches. Other E-FT64983* individuals in Western-Central-Eastern Europe, Italy
R-M207>M173>M420>M459>CTS5437>M515>M198>M417>PF6162>Z93>Z94>Z2124>Z2125>Z2123>Y934>Y7094>YP4931>**BY61693**	R1a1a1b2a2a1d6	1%	Upstream lineage R-YP4931 found in Medieval Hungary (AHP41). R1a-Z93 associated with Pontic-Caspian steppe nomadic cultures known as the Scythians	STR matches from Karpathos and Rhodos islands in the Dodecanese. Downstream lineages in Portugal and Germany

Terminal subclade in bold; asterisk (*) indicates that terminal subclade is unresolved.

**Fig. 4:**
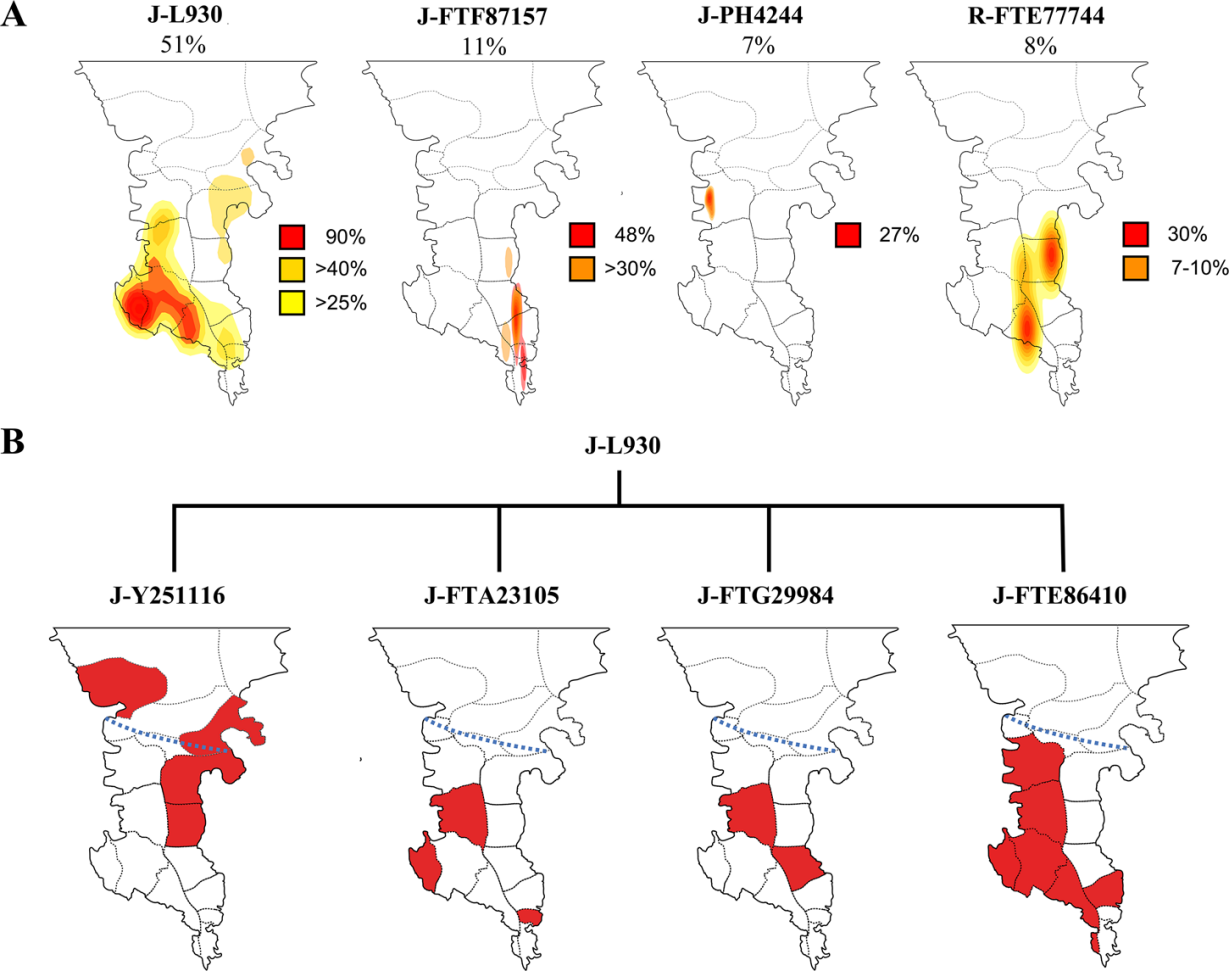
Frequency and geographical distribution of the principal Deep Maniot Y-DNA haplogroups. **A** Frequency of Deep Maniot patrilines in the different traditional subdivisions of Deep Mani. **B** Phylogeny of haplogroup J-L930, and the spatial geographical distribution of its subclades in the different traditional subdivisions of Deep Mani and adjacent regions. Note that the distributions of different J-L930 subclades are often geographically localised and non-overlapping.

**Fig. 5:**
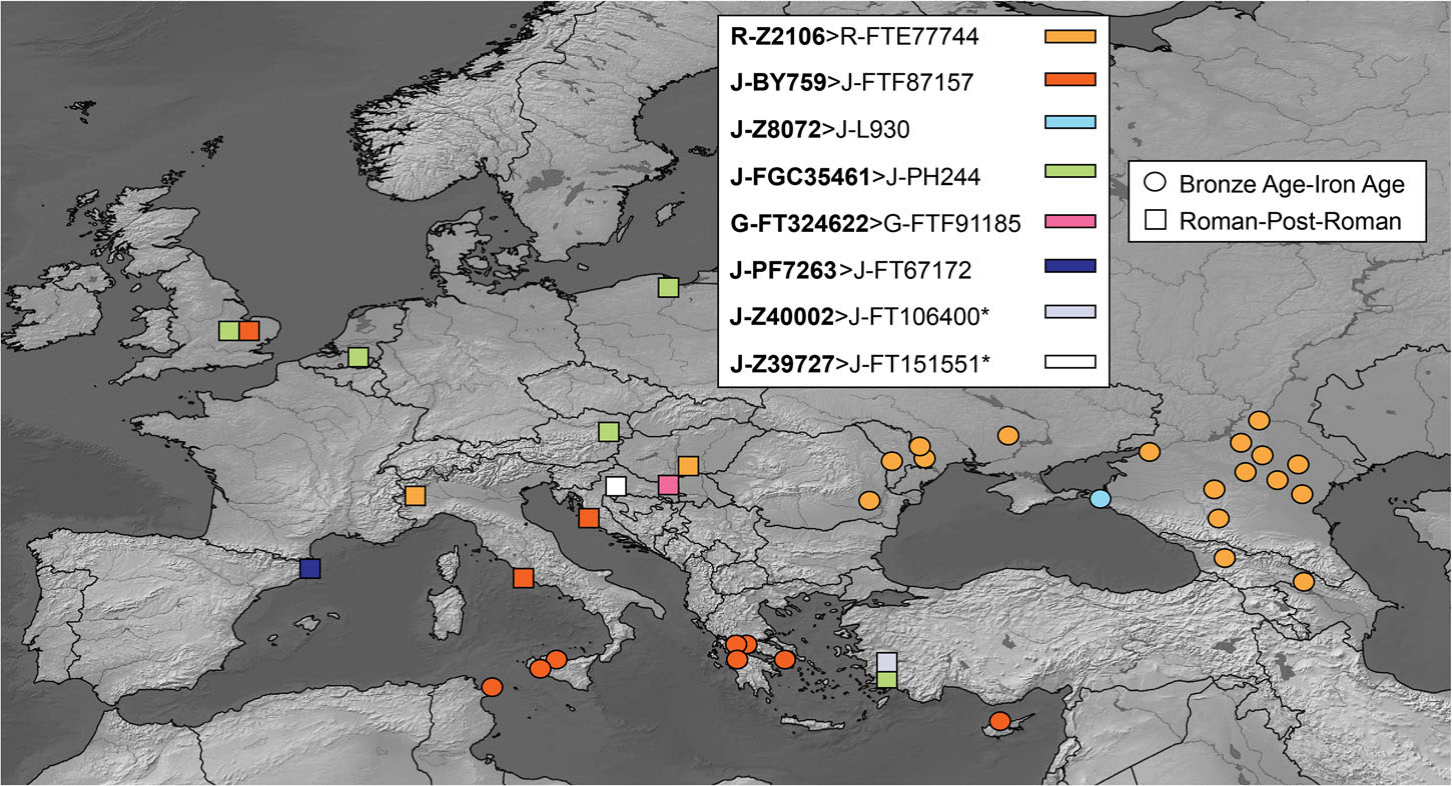
Distribution of haplogroups that are directly upstream from the most frequent Deep Maniot lineages, as identified in the ancient DNA record. Subclades shown in bold represent lineages for which ancient DNA samples have been found downstream. Note that subclade R-Z2106>R-Z2109 is excluded from this analysis. Map generated with SimpleMappr^158^.

Haplogroup J-SK1363>J-BY759>J-FTF87157 represents 11% of the Deep Maniot patrilines in our dataset. It has so far been found exclusively in the tip of the Mani Peninsula (and nowhere else globally), where it represents the majority of paternal haplogroups (48%) (Fig. 4A; Supplementary Data 2). This lineage and its parent branch J-BY759 are strongly associated with Bronze and Iron Age Greece^35,36^, where they account for 15% of Mycenaean and archaic Greek patrilines (Supplementary Data 3). J-BY759 and its daughter branches have also been found in Greek colonies in Bronze Age Cyprus^33^ and Sicily (especially Doric sites)^79^, in individuals from Punic colonies with an Aegean Bronze Age autosomal profile^80^, and in Imperial Rome^38^ (Fig. 5). Upstream branch J-SK1363 and its numerous daughter lineages have been recorded in Middle Neolithic-Chalcolithic cultures from present-day Bulgaria^81^, Croatia^82,83^, Hungary^84^, Romania^36^ and the Cycladic Culture of the Aegean^85^, confirming the presence of this lineage in the Balkan region and the Aegean since at least 6,500 BCE. Based on the above, Deep Maniot haplogroup J-FTF87157 indicates direct descent from the inhabitants of Bronze Age Greece, whose present-day expansions are confined to the Mani Peninsula (Fig. S1).

A subset of the newly genotyped Deep Maniots and a Sicilian from Palermo (retrieved from the FamilyTreeDNA Y-STR and Y-SNP database) form a novel branch of R1b-Z2103>Z2106 (R1b1a1b1b3), namely R-FTE77744 (8% frequency in Deep Mani, Fig. 4A). Haplogroup R1b-Z2103 (R1b1a1b1b) was the predominant paternal lineage of the Yamnaya culture from the Pontic-Caspian steppe, which is associated with the dissemination of Indo-European languages into the Balkans and Armenia during the Early Bronze Age (EBA)^33,36,86,87^. Although the ultimate origin of R-FTE77744 clearly lies in the EBA Pontic-Caspian steppe, its more recent origins are puzzling. R-Z2106 has three daughter branches with ancient and present-day samples found almost exclusively in the North Caucasus (Fig. 5), one branch (R-BY44400) in EBA Moldova, Romania, and Ukraine, and another branch, R-Z2108/Z2109, is found overwhelmingly in the Bronze Age cultures of Albania, Greece, and North Macedonia^33^ (Fig. S2). No STR or SNP matches for R-FTE77744 were found in the FamilyTreeDNA database (>995,000 individuals) and an extensive dataset of populations from the Caucasus (Supplementary Data 4). Although R1b-Z2103>Z2106>Z2108/Z2109 is the predominant steppe-related patriline in EBA-IA Greece, other R1b haplogroups have also been found (R1b-PF7563, R1b-V1636, Supplementary Data 3), suggesting considerable diversity of Eneolithic-EBA Pontic-Caspian steppe-derived lineages in ancient Greece. It is therefore likely that R-FTE77744 is yet another, remarkably rare, relict lineage from EBA-IA Greece. This hypothesis is further supported by the split between Deep Maniots and the Sicilian individual carrying this subclade, which is estimated at ca. 810 BCE—concurrent with the earliest phase of Greek settlement in Sicily during the Iron Age^88^.

The origins of the remaining Y-DNA haplogroups of Deep Maniots are often obscure, although, as we show in Table 1 and S1 Text, a West Asian or Balkan origin during the Roman Period or earlier is likely for almost all of them.

We define haplogroup J-L26 (J2a1a)>J-PF5087>J-PF5160>J-L930 as the Deep Maniot Modal Lineage, due to its extraordinarily high frequency across Deep Maniots (51%; Fig. 4A), and near exclusive association with this population. In FamilyTreeDNA’s dataset of 673,000 SNP-tested users, only 8 individuals with haplogroup J-L930 and no known Deep Maniot ancestry were located (Supplementary Data 7). Of these, 7 share large (12–43 cM) identity-by-descent (IBD) segments with two autosomally tested Deep Maniots in our dataset, suggesting a recent origin from Deep Mani (Supplementary Data 7). For reference, the two autosomally tested Deep Maniots share 26–62 cM of their ancestry with genealogically unrelated Deep Maniots in FamilyTreeDNA’s autosomal (Family Finder) database (Supplementary Data 7). Furthermore, as noted above (Fig. 3), all J-L930 STR-based matches from FamilyTreeDNA’s database also share recent (1300–1600 CE) matching with Deep Maniots, further showcasing the association of this lineage with the Mani Peninsula.

**Fig. 6:**
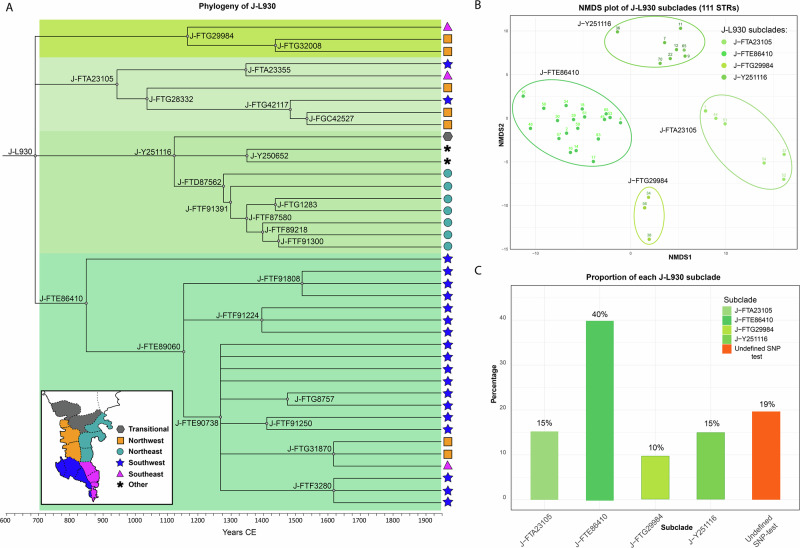
Phylogeography of the main Deep Maniot patriline, Y-DNA haplogroup J-L930. **A** Phylogenetic tree of J-L930 based on 41 Y-DNA sequences (derived from FamilyTreeDNA’s manually curated Y-chromosome DNA haplotree)^66,95^. Bottom left inset shows main geographical zones of Deep Mani; symbols represent the geographical origin of each sampled individual. The asterisk (*) refers to a Cytherian islander and an unidentified sample from the Harvard Personal Genome Project. **B** NMDS plot generated from 111 STR values from the main subclades of J-L930. Each point represents an individual sample, with the sample number indicated above the corresponding dot (see Supplementary Data 1 for full sample details). **C** Frequency of each J-L930 subclade. Note that 19% of the results correspond to low-level SNP testing, which does not allow for final subclade resolution.

By reconstructing the phylogeny of J-L930 (Fig. 6A), we show that it has four daughter branches with remarkably localised distributions, namely: J-FTE86410, the most frequent subclade, which dominates the area of Katopangi and Niklianiko (southwest Deep Mani); J-FTG29984, largely found in the area of Xoumero (northwest Deep Mani); J-FTA23105, primarily found in Xoumero-Katopangi; and J-Y251116, which is exclusively found in eastern (Prosiliaki) Deep Mani, with the exception of an islander from Cythera, ca. 40 km off the coast of Eastern Deep Mani—an island known to have received Deep Maniot settlers^12^, and a single person from the transitional area between Deep and Outer Mani. These branching patterns are also mirrored in an NMDS plot of 111 Y-STRs of all high-level tests of J-L930 (Fig. 6B). Therefore, three of the four daughter branches of J-L930 are found in western (Aposkieri) Deep Mani, and the earliest splitting subclade, J-FTE86410 (ca. 860 CE), experiences all its branching there, where it also attains its highest frequency (Fig. 6C). Considering these branching patterns, we propose western Deep Mani as the likeliest geographical area for the origin of J-L930.

Despite its present-day abundance in Deep Mani, the distant origins of J-L930 are puzzling, as it has never been found in the aDNA record. Branching patterns of parallel lineages to J-L930 in FamilyTreeDNA’s haplotree may indicate an association with the Neolithic and EBA Levant and Cyprus, although parent branch J-Z8072 (at 7500 BCE) has been found in BA North Caucasus (1450–1200 BCE)^89^ (Fig. 5), potentially indicating an origin in that region. Additionally, a present-day individual from Albania is also known from upstream lineage J-FGC68843/J-Y31950 (at 2800 BCE)^67^, indicating possible connections with the EBA Balkans.

Overall, virtually every Y-DNA haplogroup found in Deep Mani reveals a consistent pattern of paternal ancestry rooted in the ancient Balkans and West Asia (Table 1, Supplementary Data 8, 9), with strong associations to Bronze Age, Iron Age, and Roman-period Greek-speaking populations (S1 Text, Table 1). These lineages, while diverse in origin, are united by their extreme rarity outside Deep Mani and their apparent arrival prior to or during Antiquity. The haplogroup composition of Deep Maniots suggests the presence of longstanding isolation and localised expansion within different regions of Deep Mani, resulting in the frequency patterns observed in the present day.

### Present-day Deep Maniots descend from founders who lived during the 4^th^−8^th^ centuries CE

The Y-DNA data presented here, together with previous autosomal analyses^31^, have identified Deep Maniots as a distinct population isolate. However, when the Deep Maniots arose as a distinct group has remained unanswered.

**Fig. 7:**
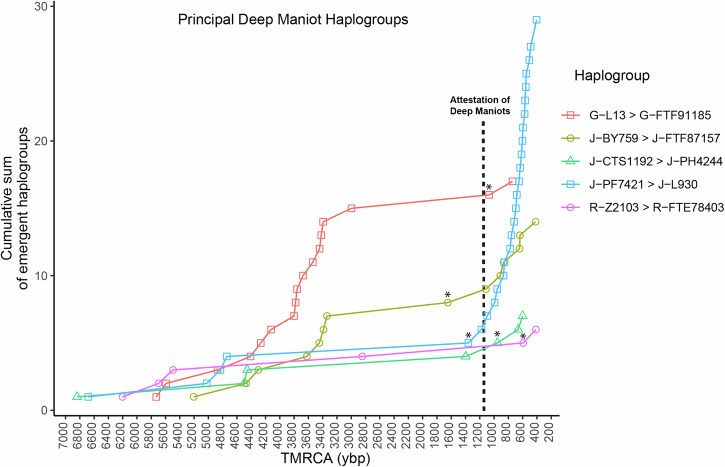
Graphical representation of clade formation (cumulative sum of new subclades) of the principal Deep Maniot Y-DNA haplogroups, using TMRCA estimates from the manually curated FamilyTreeDNA haplotree^95,110^, which is generated using targeted enrichment data (700 STRs and 750,000 SNPs). An asterisk indicates the date during which the Deep Maniot-specific mutation arose for each of the plotted haplogroups. Graph based on Supplementary Data 10.

To gain insights into the temporal origins of present-day Deep Maniots, we plot the mean TMRCAs of haplogroups specific to this population for which extensive data is available (J-L930, J-FTF87157, R-FTE77744, J-PH4244) (Fig. 7; Supplementary Data 10). Remarkably, the two most frequent Deep Maniot haplogroups (J-L930, J-FTF87157, 62% of all patrilines) show a sudden and steep increase in subclade diversity only after 380–670 CE (Fig. 7), suggesting that this was the beginning of a period of population expansion, after a significant bottleneck. A similar date was provided for J-L930 by Yfull (ca. 800 CE, branch name J-Y239616)^67^, despite employing a different method of TMRCA estimation^90^ and using a much smaller dataset (*n* = 4). These results are consistent with the first mentions of the Bishopric of Mani (901–907 CE), the appearance of Deep Maniots as a distinct group in historic records (ca. 950 CE)^13^, and putative dating of the earliest megalithic constructions in the Mani Peninsula (ca. 8^th^ century CE)^23^. Moreover, the branching patterns of the two most frequent Deep Maniot haplogroups, J-L930 and J-FTF87157, can also provide crucial insights into the settlement dynamics of the Mani Peninsula, as they reveal striking geographic structuring that may reflect historical migrational waves and founder effects within Deep Mani (Fig. 6A, S4; S1 Text).

### A chronology of the Deep Maniot clan system

The clan system of Deep Mani is one of the major societal characteristics of this population, which differentiates them from all other mainland Greeks^11,12^. However, the origins of the Deep Maniot clan system remain obscure, with one major study suggesting an origin in the mid-16^th^ century^11^.

**Fig. 8:**
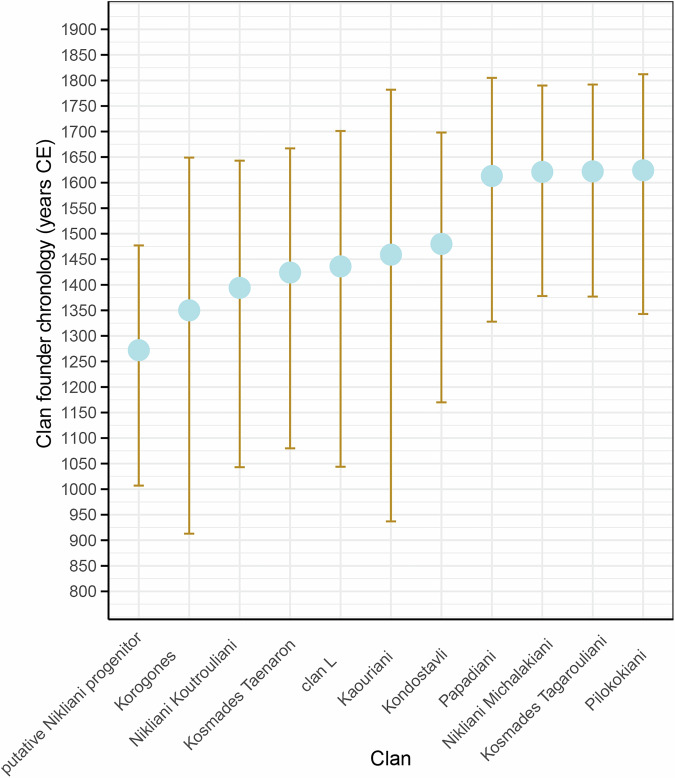
Y-DNA haplogroup TMRCAs between two or more men belonging to Deep Maniot clans, as recovered by FamilyTreeDNA’s haplotree^66,95,110^. The analysis is based on targeted enrichment sequencing, incorporating ~700 Y-STR markers and 750,000 SNPs^59^.

Our sampling strategy included testing of major Deep Maniot clans, typically inhabiting different villages and reporting shared patrilineal descent (Supplementary Data 1). In 11 of these cases, our dataset includes two or more individuals, which enables us to provide a minimum estimate for the chronology of their founders (Fig. 8; Supplementary Data 11). Our analysis suggests that founders of key Deep Maniot clans lived between 1350–1600 CE (Fig. 8), at least 200 years earlier than their suggested first appearance by previous historical studies^11,12^. These dates agree with earlier historical sources, which indicate the destruction of tower houses in Mani by the Imperial administration in 1415 CE, in order to restore order in the region^91^. Similar tower houses, which are integral to the institution of the clan, were described in 1445 CE by travellers to Deep Mani^92^. Together, these findings offer the first genetic framework for dating the emergence of the Deep Maniot clan system, revealing a timeline that opens new avenues for understanding the sociopolitical transformations that shaped Deep Mani during the Late Medieval period. We present detailed information for the remarkable oral and genetic history of each analysed clan in S1 Text.

Kinship is only one of the manifestations of the Deep Maniot clan system. Another aspect of this social organisation concerns the semi-mythological origins of the founder of each clan. Among the Deep Maniot families in our dataset, we recorded 15 mythologies suggesting origins from Eastern Roman officials, Emperors, Crusaders, or high-ranking individuals from other parts of the Peloponnese (S1 Text; Supplementary Data 12). Considering that most of these individuals patrilineally descend from haplogroups that are exclusive to Deep Mani (especially J-L930, found in 11/15 of the mythological ancestors, Supplementary Data 12), we suggest that oral histories of noble descent are unsupported by our genetic findings. We stress that genetic relatedness represents only one dimension of descent, and does not encompass the full spectrum of identity, collective memory, and belonging that these origin stories convey.

### The neighbours of Deep Maniots represent a patrilineally distinct population

Considering the stark differences in Y-chromosome haplogroup composition between Deep Maniots and other mainland Greeks, the paternal ancestry of geographically adjacent, related populations might be informative on demographic events that shaped the Mani Peninsula as a whole. The inhabitants of west and southeast Taygetos, collectively known as Outer Maniots, have been shown by previous work to be autosomally closely related to the Deep Maniots, as they share large mean pairwise IBD segments (ca. 36 cM)^31^. However, they occupy distinct positions on autosomal-based PCAs and are characterised by different K8 ADMIXTURE components in the study of Stamatoyanopoulos et al.^31^, suggesting additional demographic influences compared to Deep Maniots. While both PCA and ADMIXTURE analyses can be influenced by genetic drift^93,94^, autosomal distinction is further supported by the findings of Raveane et al.^32^, who show that populations from Laconia and eastern Taygetos exhibit higher proportions of Bronze Age Pontic-Caspian steppe-related ancestry compared to Deep Maniots, possibly due to higher input from northeast European groups. By exploring the autosomal customer dataset of FamilyTreeDNA, we located paternal lineages for 13 Outer Maniots, primarily from Messenian Mani (*n* = 11) (Supplementary Data 13). Although our sample size is small, some inferences can be drawn.

The principal haplogroup of Outer Maniots is E-V13>E-Z16659 (46%), in contrast to Deep Maniots, where E-V13 is remarkably rare (1%). Remarkably, one sequenced Messenian Maniot belongs to subclade E-V13>E-Z16659>E-Y3183>E-S2972>E-PH3589>E-S2978>E-BY5285>E-BY116895>E-FTF92003, which is different to the Deep Maniot E-V13 lineage (E-V13>E-BY3880>E-Y16729>E-BY202063>E-FT64983*).

The Messenian Maniot forms a subclade with a man from Trifylia, in western coastal Messenia, dated to ca. 620 CE, while upstream lineage E-BY202063 (dated to 480 CE), is found in an individual from the Evrotas valley in Laconia, 80 km to the north of Messenian Mani^95^. The distribution and TMRCA of this lineage suggest a presence of this subclade in the broader southern Peloponnese at least since the Late Roman period. All other lineages found in Outer Mani (e.g. E-M34, G-PF3146, Ι-Μ223, J-S18579; Supplementary Data 13) are not present in our extensive dataset of Deep Maniots, even at the macro-haplogroup level. We also found two lineages associated with the Migration Period (R1a-Z282>R-YP372, I-M423>I-CTS10228; Supplementary Data 13), which account for 15% of the patrilines in Messenian Mani.

Based on the above preliminary results, the Outer Maniots represent a population that experienced different influences on paternal ancestry from their southern kin, the Deep Maniots. Considering the autosomal relatedness (based on IBD-sharing) and the similarities in social organisation and dialect of the two populations^11,12,31^, it is likely that complex demographic and genetic processes may have led to a transfer of the Deep Maniot cultural package to Outer Mani, uniting the two areas into a common zone of influence and interaction, leading to shared features in their identity.

### The origins of Deep Maniot women

Given that Deep Maniot society was strongly patriarchal until recent times^11^, the histories of its women remain largely obscure. To address this knowledge gap, we examined mtDNA sequences and terminal haplogroups in 50 Deep Maniots of our dataset whose maternal lineages originate from the Mani Peninsula (Supplementary Data 14), by querying FamilyTreeDNA’s Mitotree (>260,000 present-day and 10,800 aDNA samples) and GenBank.

**Fig. 9:**
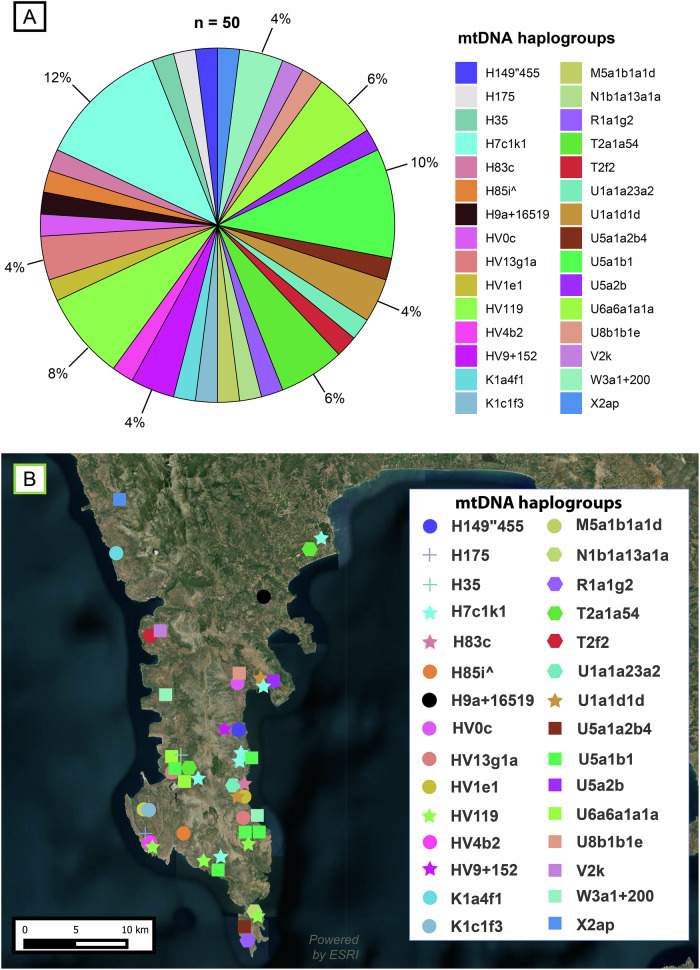
Maternal lineages in Mani. **A** mtDNA haplogroup frequency in Mani. **B** Geographical distribution of mtDNA haplogroups in the Mani Peninsula. The satellite image of the Mani Peninsula was created with QGIS 3.40.0.48^155^ using a basemap from the Esri World Terrain Base Map, ArcGIS Online (Sources: Esri, USGS, NOAA)^156,157^.

Our analysis revealed the presence of at least 30 distinct maternal haplogroups in Deep Mani and the transitional zone of Oetylon and Gytheion (Fig. 9; Supplementary Data 14).

Although the limited resolution of mtDNA renders the origins of many of Deep Maniot matrilines obscure, distinct connections with aDNA samples can be made for most maternal lineages. Haplogroups H7c1k1, H35, HV1e1, HV4b2, HV119, N1b1a13a1a, U1a1a23a, U1a1d1d, U5a1a2 and U8b1b1e, collectively accounting for 38% of all matrilines, have broad connections to BA-IA populations from the Balkans, West Asia, Caucasus, and the Levant, based on their matches to ancient and present-day populations (Supplementary Data 14). We mention the phylogeography of certain region-specific lineages below.

**Fig. 10:**
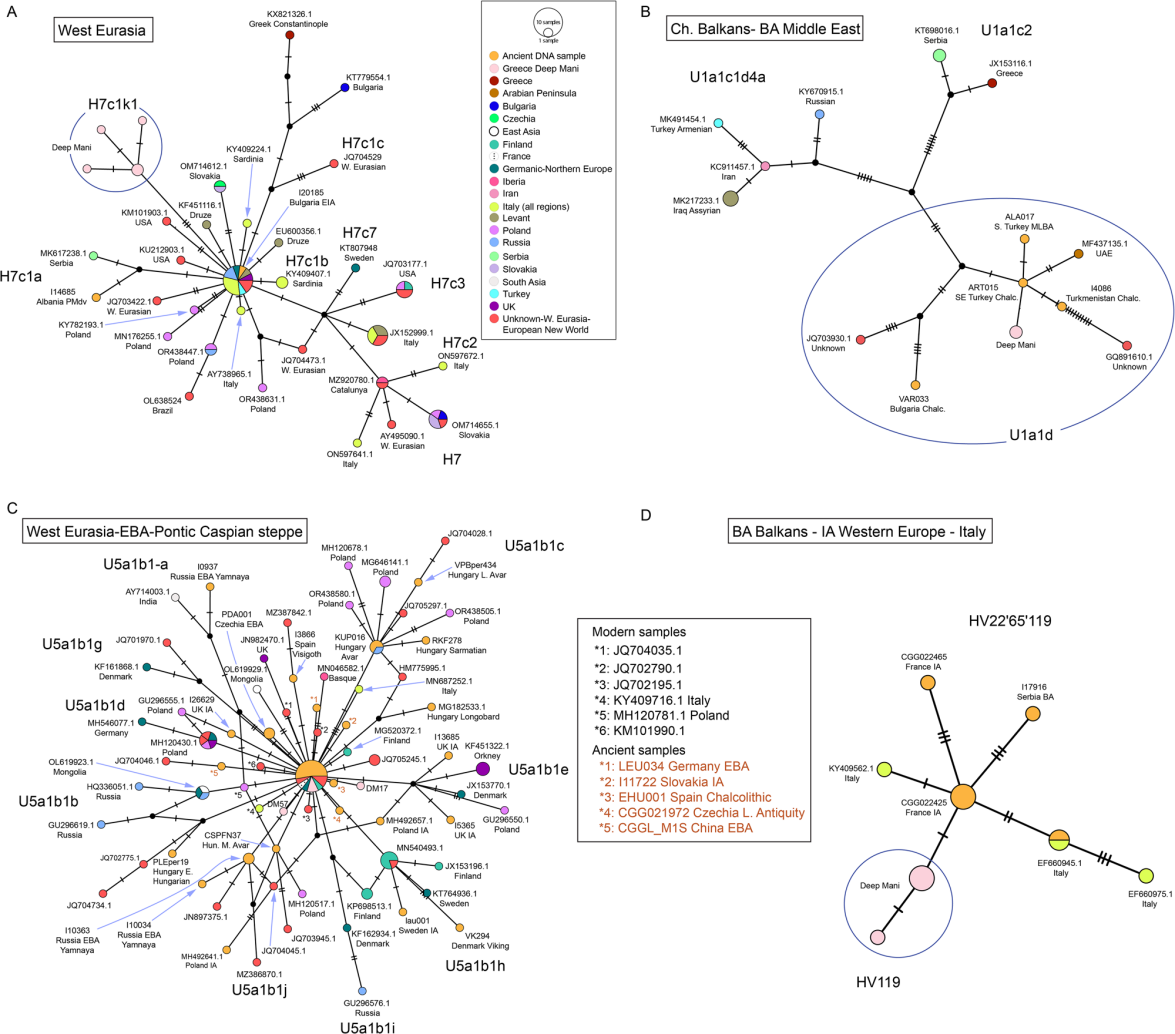
Median joining networks for haplogroups H7c, U1a1, U5a1b and HV22'65'119-HV119, obtained from complete present-day and ancient mtDNA genomes. **A** Network of haplogroup H7c. **B** Network for haplogroup U1a1. **C** Network for haplogroup U5a1b. **D** Network for haplogroup HV22'65'119-HV119; panel slightly enlarged due to the smaller number of analysed lineages compared to the other haplogroups.

The origin of H7c1k1 (12%) likely lies in the ancient eastern Mediterranean, as H7c1 lineages have been found in the BA, Roman and Medieval Balkans^36,39^ (Fig. 10A) and present-day matches include a Romanian and two Saudis, while upstream lineage H7c1k is found in a present-day Lebanese individual (Supplementary Data 14).

Haplogroup H35 (2%) is a particularly rare lineage that in the aDNA record has been found exclusively among individuals whose autosomal profile is similar to populations from Early Iron Age Bulgaria (Supplementary Data 14). Individuals with this autosomal profile and maternal lineage have been found in Iron Age North Macedonia and Moldova, as well as Late Antique Italy and Hungary (Supplementary Data 14). We therefore interpret this lineage as originating from the Iron Age Balkans.

Lineage U1a1d1d (4%) has a particularly old presence in the Eastern Mediterranean, having been recovered from remains in Chalcolithic Bulgaria and southeastern West Asia (Fig. 10B).

HV119 (8%) and its parent haplogroup, HV22'65'119, represent an extremely rare lineage, with ancient samples from Bronze Age Serbia, Iron Age northeastern France, an undated ancient individual from Tarquinia, and present-day matches from Italy (Fig. 10D), Slovenia, Germany and Holland (Supplementary Data 14). This haplogroup likely represents a lineage that ultimately originates from western Europe, especially the alpine region and the Italian Peninsula, which may have entered the Balkans already by the Bronze Age.

Two lineages, R1a1g2 and K1a4f1 (4%), show strong connections to ancient and present-day populations of the Caucasus (Supplementary Data 14).

Some lineages in Deep Mani have a broad West Eurasian distribution that does not currently permit their association with specific populations after the Bronze Age—these include T2a1a54, U5a1a2b4, U5a1b1, U5a2b, W3a1 + 200, and K1c1f3 (totalling 24% of all matrilines). Although T2a1a54 has been found only in Deep Maniots and two present-day Greeks, T2a1 and its subclades are known from a broad range of aDNA samples and appear to have originated in the Pontic-Caspian steppe with the Yamnaya and other Indo-European groups that followed them in the region (Supplementary Data 14). U5a1b1 (10%) can also be traced to the Yamnaya steppe herders, with daughter lineages across present-day West Eurasia (Fig. 10C).

A group of haplogroups, namely, H9a + 16519, HV9 + 152, H149″455, T2f2, X2ap (collectively accounting for 12% of all matrilines) are lineages that so far make their appearance in the Balkan aDNA record during the Migration Period, in present-day northeastern Europeans, or, in the case of T2f2, in a Roman Period individual with a nomadic steppe profile (Supplementary Data 14).

Some surprising connections to more distant populations have been observed. Haplogroup U6a6a1a1a (6%) originates from the indigenous populations of the Maghreb and the Canary Islands^96–98^, while M5a1b1a1d (2%) represents one of the founding Roma lineages^99^.

A group of haplogroups (H175, H83c, H85i^, HV0c, HV13g1, V2k; 14% of all matrilines), do not have sufficient phylogeographic resolution to elucidate their origins based on current aDNA or present-day distributional patterns.

## Discussion

In the present study, we unveiled the uniparental inheritance patterns and ancestry of Deep Maniots for the first time. In agreement with previous research exploring autosomal ancestry^31^, we show that Deep Maniots represent a genetic island within mainland Greece. Although the previous study suggested limited contribution of Slavic peoples to the ancestry of present-day Deep Maniots, it did not explore the deeper origins of the Maniots or their relationship to ancient populations. Our findings demonstrate that Deep Maniots overwhelmingly descend from paternal lineages associated with the populations of BA-IA and Roman Greece. Remarkably, the complete absence of haplogroups associated with Germanic, Slavic, Aromanian, Albanian, and Western European populations, which contributed (to varying degrees) to the ancestry of mainland Greeks over the past 1400 years (Fig. 2A, B), further supports historical accounts that Deep Maniots were largely shielded from the tumultuous demographic transformations that took place in the Balkans during the Migration Period and for centuries to come, up to the present.

We note that the pronounced founder effects observed in Deep Maniots and the near absence of Y-STR matches with non-Deep Maniots in our dataset are key indicators of genetic isolation, very likely associated with drift^78^. In particular, the high frequency (51%) of J-L930—which we name the Deep Maniot Modal Lineage, and J-FTF87157 (11% frequency), coupled with their restricted geographic distribution, suggest that genetic drift has probably amplified certain lineages in Deep Mani while eliminating others over time. This process likely contributed to the Deep Maniots’ genetic distinctiveness relative to other mainland Greeks.

However, although genetic drift has likely shaped the Deep Maniot genetic landscape, particularly in terms of haplogroup frequencies, multiple lines of evidence suggest that the ancestry of Deep Maniots is not the result of merely stochastic processes. While drift likely elevated certain haplogroup frequencies and reduced others, its effects should be broadly distributed; the absence of Migration Period lineages suggests they were likely rare or absent in the founding Deep Maniot population. Furthermore, an autosomal study has shown limited influence from present-day Slavic populations^31^, while in PCA analyses with ancient samples, Deep Maniots cluster with the East-Mediterranean-shifted Imperial Roman individuals^32^, close to Mycenaeans, suggesting affinity to pre-Slavic southern Balkan^3^ and Italian populations^37^ with similar genetic profiles. Taken together, these findings support our interpretation that Deep Maniots represent a snapshot of regional genetic diversity prior to the demographic transformations of the Migration Period, retaining a significant proportion of ancestry dating back to at least the Late Roman era.

While the observed founder effects in Deep Mani, dated to the 4^th^–7^th^ centuries CE, most likely suggest continuity from earlier local populations, introgression from neighbouring regions of individuals with a Roman Period autosomal profile, followed by re-expansion could be suggested as an alternative explanation. Haplogroup J-L930, for instance, which expands during and after the 7^th^ century CE, could reflect such a process. However, the absence of J-L930 from global Y-DNA datasets suggests its directly ancestral lineages are either extremely rare or even extinct outside Deep Mani. Furthermore, the high frequency of J-L930 in our dataset may have obscured finer-scale variation among other Deep Maniot lineages, which were less likely to be encountered during our sampling. Additionally, Mani’s tradition of blood feuds may have led to localised lineage extinctions, reducing Y-DNA diversity and erasing lineages that once captured broader haplogroup variation—including earlier branching of J-L930 within Deep Mani. Taken together, these patterns likely reflect repeated cycles of expansion and contraction within an insularised population, shaped by internal dynamics rather than external input.

The abovementioned information also enables us to add crucial insights into a vexing question regarding the origins of the Deep Maniots, that is, whether they descend from earlier populations in the Mani Peninsula, or from Greek speakers who sought refuge there from other regions of the Empire during and after the Migration Period. While this question cannot be conclusively resolved without an extensive aDNA transect of Roman and Medieval Deep Mani, our findings point to genetic continuity from local groups, supported by two compelling lines of evidence.

Firstly, the temporal analysis of the most frequent Deep Maniot patrilines, J-L930 and J-FTF87157, reveals a pronounced founder effect occurring between 380 and 670 CE, likely following a population bottleneck. This genetic signature aligns with the earliest historical references to Deep Maniots as a distinct Greek-speaking people^18^ and implies that the foundational paternal lines of this community stem from individuals who endured this pivotal bottleneck event as part of a single, cohesive group. It is therefore very likely that most present-day Deep Maniots derive their paternal ancestry from the 4^th^–8^th^ century inhabitants of Deep Mani.

Secondly, should Deep Maniots descend from an amalgamation of different Greek speakers from nearby or more distant regions over the past 1400 years, one would expect STR and SNP-matching with other populations. The exceptional rarity of most Deep Maniot patrilines outside the Mani Peninsula and the absence of matches with other populations are clear indicators of longstanding isolation that likely predates the Migration Period. In particular, the deep split of J-FTF87157 within Deep Mani (380 CE) predates the 6^th^ century CE invasions and settlements of Slavic peoples into the region of present-day Greece^2,3^, indicating long-term continuity of Deep Maniot lineages within the Mani Peninsula.

The founder effect that defines the two most frequent Deep Maniot patrilines (J-L930, J-FTF87157) around 380–670 CE may have had multiple causes. Key historical stressors during this period include the Justinianic Plague (6^th^–8^th^ centuries CE)^2,6,30^, the onset of Slavic migrations into Greece (ca. 580 CE)^2,4,100^, and a series of Arab maritime incursions affecting the Greek islands and coastline post-650 CE^7,30^. Each of these events could have reinforced the isolation—both cultural and genetic—of Deep Mani’s inhabitants and may have even caused a dramatic reduction in the size of the population. Notably, the four-century-long silence in historical sources concerning Deep Mani and its people likely marked a formative period in which the Deep Maniots developed into a distinct group, giving rise to enduring traits such as their unique megalithic building traditions.

Indeed, one of the more striking revelations of our research lies in the interplay between material culture and genetics. The distribution of Mani Peninsula’s megalithic architecture—largely dated to the pre-Christian or early Christian era^22^—precisely mirrors both the historic borders of Deep Mani (south of Areopolis and Skoutari) and the geographical prevalence of Deep Maniot-specific paternal lineages (Fig. S5). In contrast, regions immediately north of Deep Mani exhibit distinct patrilineal compositions, implying divergent demographic histories. This close correspondence suggests that the megalithic structures were the cultural artifacts of a population directly ancestral to today’s Deep Maniots—one that remained bound within this unique cultural zone for centuries, if not millennia.

Due to their isolation and the diminished role of imperial administration in this corner of the southern Peloponnese, the Deep Maniots developed a unique system of customary law, which involved blood feuds as a last resort in conflict resolution. The Deep Maniots’ aptitude for warfare is documented as early as the 13^th^ century, when the medieval feudal Latin states, specifically the Principality of Achaea, attempted to quell their rebellious activities by building fortresses and castles along the northern borders of Deep Mani^10,24^. After the Eastern Roman Empire regained control of the Peloponnese from the Latin states, many defensive fortresses or tower houses in Mani were allegedly destroyed in 1415 CE, in order to prevent anarchy and eliminate warfare practices used by the Deep Maniots^10,24,91^.

Such historical events point to a militarised culture that likely facilitated the development of clan structures. Indeed, our ability to trace the founders of certain Deep Maniot clans to the 14^th^-15^th^ centuries CE supports this hypothesis, complementing broader scholarship, which suggests that clan institutions often emerge in regions lacking centralised governance, in order to foster cooperation in challenging circumstances^11,101–103^. Although previous studies suggested an origin of the clan system in the 16^th^ century^11,12^, our analyses have recovered clan founders two centuries earlier (Fig. 8). This result should be treated as a minimum estimate, as examination of censuses^12,104^ and Deep Maniot oral traditions (S1 Text) demonstrate constant turnovers in clan distribution in the Mani Peninsula. Indeed, we show that the earliest recorded clans of Deep Mani, who were prominent and widespread in 1514, occupy only a handful of locations in the present day (S1 Text). It is therefore likely that clans existed prior to the 14^th^ century and may have become reduced or even extinct before the earliest censuses took place.

Our analyses of the maternal ancestry of Deep Maniots revealed a more complex genetic landscape, with influences ranging from the ancient Balkans and the Levant to Western Europe and the Maghreb. We should highlight, however, that mtDNA haplogroups provide lower resolution for population genetics compared to Y-chromosome haplogroups, due to higher mutation rates and smaller genome size, leading to backmutation and saturation^105^. As a result, our analyses can help elucidate the phylogeographic origins of Deep Maniot maternal lineages, but in most cases, the arrival date of each mtDNA haplogroup to Deep Mani cannot be determined with confidence.

Although the precise timeframe during which many maternal lineages entered the Deep Maniot gene pool cannot be established for all lineages, it is likely that a substantial portion was already present in the founding population. Mirroring overall Y-DNA frequency patterns, several maternal haplogroups, namely H7c1k1, HV119, T2a1a54, U5a1b1, and U6a6a1a1a appear to have undergone founder effects, collectively accounting for ~42% of all matrilines (Fig. 9A). Notably, FamilyTreeDNA’s mtDNA Time Tree estimates the TMRCA for the HV119 and H7c1k1 founder events to fall between ca. 540 CE and 866 CE, a period that overlaps with the Y-DNA founder-effect timeframe (380–670 CE).

As with Y-DNA, several maternal lineages are Deep Maniot-specific, with most showing no close matches to other populations and a distribution confined to the Mani Peninsula. Notably, haplogroup H7c1k1 may represent the maternal analogue of J-L930, as it is found across Deep Mani and extends to several locations in Outer Mani as well (Fig. 9B). We also did not find any non-Deep Maniot matches to H7c1k1 other than a single Romanian and two Saudi Arabians (Supplementary Data 14).

We should also note that mtDNA lineages of 5 Neolithic samples from Diros Cave in Deep Mani have been previously published^83^ (Supplementary Data 3). None of these haplogroups have been found in the present-day population of Deep Mani (Fig. 9), which is attributable either to limitations of the sample size of our dataset (*n* = 50), or to uniparental turnovers during the Neolithic-Bronze Age transition and later periods.

Despite these general patterns of isolation, we also detected limited maternal gene flow from non-Deep Maniot populations. We recovered a single M5a1b1a1d sample—a lineage commonly associated with Roma populations, who are known to have arrived in the Balkans by at least the 12^th^ century CE^106^—as well as haplogroups likely linked to the Migration Period (H9a + 16519, HV9 + 152, H149″455, T2f2, X2ap). In the context of a patriarchal and kinship-oriented society such as that of the Deep Maniots, the integration of a limited number of foreign individuals may have been facilitated primarily through women.

Overall, our study demonstrates that Deep Maniots overwhelmingly descend paternally from ancient Greeks and Eastern (Greek-speaking) Romans known today as the Byzantines. This truly remarkable phenomenon may stem from the unique sociocultural dynamics of Deep Maniots and the geography of the Mani Peninsula, which led them to isolate themselves from different waves of migration, settlement and integration taking place in the surrounding regions. As a result, this hardy group represents a snapshot of the genetic landscape of the Greek-speaking world prior to the demographic turmoil of the Migration Period. While we anticipate that the quest for the origins of Deep Maniots will undoubtedly continue, our work provides a fundamental framework that can inform the interpretation of archaeological, historical, anthropological and linguistic history of the Greek-speaking world and southeastern Europe more broadly.

## Methods

### Study setting and sampling strategy

We recruited Deep Maniot participants through two complementary approaches:

### 1. Field-based recruitment and new DNA sampling

We collected saliva samples from 75 Deep Maniot volunteers, each with uninterrupted patrilineal ancestry from villages across the entire geographic range of Deep Mani. Of these, 68 individuals underwent next-generation targeted enrichment sequencing, while 7 were genotyped for Y-STRs (using 37 and 111 loci). Additionally, 50 participants also reported matrilineal descent from Deep Mani, enabling analysis of their mtDNA. We focused on individuals originating from settlements that have been continuously inhabited for at least the past 500 years. Given that traditionally, Deep Maniot villages were settled by a single or a handful of clans^11,15^, we ensured sampling of genealogically unrelated clans per settlement wherever possible, as well as clan-less families, if these were present. As a result, our sampling strategy provides a comprehensive and representative dataset of the Deep Maniot genetic landscape. Given that many settlements in Deep Mani no longer support a permanent population and due to the large-scale emigration of Maniots in the 20^th^ century, more than half of the study’s participants comprised members of the Deep Maniot diaspora.

Our study follows a community-based participatory research (CBPR) approach incorporating research, reflection, and action in a cyclical process between researchers and participants. The Deep Maniot community plays a central role in our research, with participants engaged in every aspect of the research process. Prior to their inclusion in the study, all newly sequenced participants were informed in non-scientific language about the study’s aims and provided informed consent for use of their data for the purposes of strict scientific enquiry. We highlighted that participation in this study is completely optional. Individuals would not receive any financial or material rewards for their involvement and they were free to opt out if they wished to do so. Some participants requested that their clan or subclan name be included in the study, a few opted for it to be anonymised, while others did not want to provide their clan’s name. We note that any clan name similarities with non-Maniot Greek surnames are the result of synonymy and does not correspond to any genealogical or genetic relationship with our study’s participants.

Together with the DNA samples, we also collected personal data from newly sequenced individuals solely to facilitate communication with each volunteer, to inform them about their results (in case they opted to receive them), and to involve them in the study’s design. To this end, we designed and implemented a robust data management plan that complied with the General Data Protection Regulation (GDPR), as well as FamilyTreeDNA’s strict policies that ensure tester privacy and security^107^. In this way, the results of our research were communicated to the participants prior to submission of the study. This approach allowed us to engage in discussions with the participants, which informed the research questions addressed in this work. Furthermore, oral traditions of descent, kinship, and migration of each participant’s patriline and matriline were shared with us during informal discussions with the relevant community members and were combined with relevant data from the literature^5,11,15,16,108^. A certificate with a detailed explanation of the results of their personal Y-DNA and mtDNA analysis was provided to participants who requested it. These participants were given the contact details of our team should they have further questions regarding the interpretation of their results.

We maintain ongoing communication with clan members to ensure that the local community is fully informed about all the scientific articles we publish. An accessible booklet summarising the results of this study will be provided free of charge in Deep Mani.

### 2. Database recruitment

Two of the Deep Maniot volunteers also opted for the Family Finder autosomal genealogical service^109^, which uses long (>6 cM) IBD segments shared with individuals in FamilyTreeDNA’s consumer database to find genealogical connections from across the world, and to provide preliminary uniparental haplogroup determinations for more than 673,000 SNP-tested users. By querying FamilyTreeDNA’s private autosomal and STR datasets, we were able to report Y-chromosome haplogroup frequencies for 27 additional individuals with confirmed Deep Maniot origin, bringing the total number of analysed Deep Maniots to 102 (Supplementary Data 1). Among these, 17 had undergone autosomal testing, 7 were genotyped using Y-STR panels (ranging from 12 to 111 markers), and 3 had performed targeted enrichment sequencing and, after providing consent, shared their terminal subclade. We also report haplogroup frequencies for 13 Outer Maniots, an adjacent population with IBD links to Deep Maniots^31^, and two Peloponnesians belonging to an E-V13 subclade shared with Outer Mani.

### Approvals

This work contributes to the East Mediterranean Population Isolates Study (EMPIS), a collaborative effort to characterise the genetic history of culturally and historically distinct communities across the Eastern Mediterranean, approved by the European University Cyprus Bioethics Committee. Our study on Deep Mani was additionally reviewed and approved by the Bioethics Committee of the School of Sciences, European University Cyprus (Reference: 20250213, 2025–24), the Secretariat of the University of Oxford Medical Sciences Interdivisional Research Ethics Committee (Reference: R92782/RE001), the Faculty of Nursing, National and Kapodistrian University of Athens (Reference: 159263), and the Director of the Areopolis Health Centre. The research and experimental protocols were undertaken in accordance with the principles stated in the International Declaration of Helsinki for the protection of human subjects, the ethical standards of the European University Cyprus, the University of Oxford, the National and Kapodistrian University of Athens, as well as the relevant GDPR laws of the Hellenic Republic. All ethical regulations relevant to human research participants were followed.

### High-coverage Y chromosome sequences

For the purpose of phylogenetic analysis, the study included 71 high-coverage, whole Y chromosome sequences that had not been previously documented in academic literature. All participants were informed about the study’s goals and provided informed consent for their data to be utilised in scientific research. Their sequences were obtained using the Illumina NovaSeq 6000 platform, with Y-chromosome capture performed through a proprietary protocol developed by Gene by Gene (FamilyTreeDNA) using their commercially available Big Y-700 service^59^. This service’s targeted enrichment design employs 155,000 capture probes to sequence >15 Mbp of the Y chromosome at coverage levels 35–105× in depth, depending on sample quality^51,110,111^. This test offers complete Y-chromosome haplogroup resolution, capturing 700 STRs and 750,000 tree SNPs^60,111^. Alignment was based on human reference genome GRCh38^112,113^. The sequencing procedures follow Begg et al.^110^. Haplogroup assignment for aDNA samples was completed by identifying known branch-defining variants, while less weight was given to non-private mutations identified as highly recurrent variants, and to variants occurring in problematic Y chromosome regions (e.g. the centromere, DYZ19 repeat, and Yq12 heterochromatic region)^114^.

The 71 BigY-700 samples were then incorporated in FamilyTreeDNA’s human Y-chromosome phylogeny^95^, which is generated through automated shared variant detection and manual curation^110^ and employs the Y Chromosome Consortium haplogroup nomenclature^115^. The method of phylogenetic tree reconstruction and TMRCA estimation are described in Begg et al.^51,110^ and Palencia-Madrid et al.^51^. FamilyTreeDNA’s phylogeny includes >145,000 present-day user records at high sequence resolution^59,111^, as well as every publicly available NGS-sequenced aDNA male sample of sufficient coverage. This approach allowed us to compare Deep Maniot Y-chromosomes with populations from across the globe, both past and present. These insights informed our understanding of the phylogeography and putative ethnolinguistic groups who may have contributed into the Y-chromosome ancestry of present-day Deep Maniots.

## Estimation of haplotype diversity

### Y-STR analysis

#### Within-population patrilineal haplotype sharing and haplotype diversity

To identify individuals sharing identical Y-STR haplotypes within the Deep Maniot population, multi-locus haplotypes were constructed by concatenating allele values at the 111- and 17-STR level (the most commonly used STR panel in Y-DNA studies, see haplotype matching section), into a single string for each individual. These composite haplotypes were then tabulated to determine their frequency distribution. Haplotypes observed in more than one individual were classified as shared haplotypes, indicating shared paternal ancestry.

To quantify the genetic diversity within the Deep Maniot population, we calculated Nei’s haplotype diversity (H)^116^ at two levels of Y-STR resolution: using 111-locus haplotypes (*n* = 69) and 17-locus haplotypes (*n* = 75). Haplotype diversity provides a measure of the probability that two randomly selected haplotypes from the population are different and is particularly informative for assessing genetic variation in populations with uniparentally inherited markers such as the Y chromosome.

Nei’s haplotype diversity was manually computed in R using the total number of unique haplotypes and their frequency, as identified in the step above, based on the following formula:

$$H=\frac{n}{n-1}\left(1-{\sum }_{i=1}^{k}{p}_{i}^{2}\right)$$

where *n* is the total number of individuals, *p*_*i*_ is the relative frequency of the i-th haplotype, and *k* is the total number of unique haplotypes.

The finite-sample correction factor *n/(n – 1)*, corrects the downward bias caused by finite sampling, ensuring a more accurate estimation of diversity in small datasets, such as ours.

Applying Nei’s diversity index at both marker resolutions provides complementary insights into Deep Maniots’ genetic structure, as the analysis at the 111-marker level provides a high-resolution overview of present diversity, while the 17-marker level allows for the investigation of deep shared ancestry (Supplementary Data 6).

### Within-population patrilineal genetic structure and dimensionality reduction

To investigate substructure within the predominant Deep Maniot Y-chromosome lineage, we applied NMDS to individual-level Y-STR haplotypes based on 111 loci, for haplogroup J-L930, which represents 51% of the Deep Maniot sample. Allelic data were in numeric format were transformed into a genind object using the *adegenet* package (v2.1.10)^117^, specifying haploid codominant markers. A pairwise dissimilarity matrix was computed using the diss.dist() function from the *poppr* package (v2.9.3)^118^, calculating multilocus genetic distances between individuals.

NMDS was then applied using the metaMDS() function from the *vegan* package (v2.6-4)^119^, with two dimensions (*k* = 2) and up to 200 random starts (trymax = 200) to ensure convergence. The stress value was used to evaluate the fit of the ordination, with values below 0.1 considered indicative of a good representation of the data in two dimensions. NMDS coordinates were extracted and visualised using ggplot2 (v3.4.4)^120^.

### Patrilineal haplotype matching between populations

Our comparative Y-STR dataset comprised 12,722 individuals from 56 populations, including a focal sample of 74 individuals from Deep Mani, Greece. The STRs for non-Maniot individuals were sourced from the studies of Aboukhalid et al.^121^, Alakoc et al.^122^, Albarzinji et al.^123^, Balanovsky et al.^61^, Brisighelli et al.^124^, Dogan et al.^125^, El-Sibai et al.^63^, Eskandarion et al.^126^, Ferri et al.^127^, Fernandes et al.^128^, Heraclides et al.^65^, Jankova et al.^129^, Karachanak et al.^130^, Kovatsi et al.^131^, Laouina et al.^132^, Martin et al.^133^, Moutsouri et al.^134^, Nogueiro et al.^135^, Purps et al.^136^, Robledo et al.^137^, Schurr et al.^138^, Stanciu et al.^139^, Valverde et al.^140^. and the publicly available samples of the Armenian FamilyTreeDNA project (https://www.familytreedna.com/groups/armeniadnaproject/about). To compare FamilyTreeDNA-genotyped individuals with the 56 focal populations, we applied the following conversion: GATA H4.1 + 1. We selected 17 Y-STR markers from our Deep Maniot dataset to ensure broad comparability with our large-scale reference panel of 12,647 previously published individuals, the vast majority of which were genotyped using the same marker set.

To ensure consistency, all allele values were first coerced into character strings prior to further analysis. To determine shared haplotypes (i.e. identical multilocus genotypes), the allele data were converted into a genind object using the ‘df2genind’ function from the *adegenet* package in R^117^, with the ploidy parameter set to 1 to reflect the haploid state of Y-chromosome markers. Multilocus genotype (MLG) groups were then identified with the ‘mlg.id’ function from the *poppr* R package^118^. Groups with more than one individual were considered as instances of shared (exact) haplotypes. The number of unique haplotypes as well as the distribution of group sizes were summarised, and the shared haplotype groups were exported for further evaluation and manual extraction of Deep Maniot-specific matches.

To capture near-exact haplotype matches, that is haplotypes that differ by only one repeat unit at a single STR locus, the allele data were also converted into a numeric matrix. Pairwise Manhattan distances were computed across all individuals using the ‘dist’ function in R. Haplotypes exhibiting a Manhattan distance of 1 were flagged as ‘near-identical’, implying that they diverged by exactly one repeat at a single STR locus. For each pair, the locus of variation and the magnitude of the repeat difference were recorded (Supplementary Data 6). These near-miss pairs were compiled into a table and matches corresponding to the Deep Maniot haplotypes were manually isolated (Supplementary Data 6).

### Analysis of molecular variance and pairwise genetic distances

We ran an Analysis of Molecular Variance (AMOVA) implemented in the *pegas* package (v1.3-1)^141^ in R (v4.3.2), on 17-locus Y-STR haplotypes (DYS19, DYS389I, DYS389II, DYS390, DYS391, DYS392, DYS393, DYS385a, DYS385b, DYS438, DYS439, DYS437, DYS448, DYS456, DYS458, DYS635, GATA H4), estimating pairwise *R*_ST_ distances between populations included in our comparative dataset (n populations = 56, n individuals = 12.720), accounting for the stepwise mutation model characteristic of microsatellite loci^142^ (Supplementary Data 5). Allele data were first converted to a numeric matrix representing raw repeat counts at each STR locus. Individuals were grouped by population using a factor vector. For each pair of populations, we extracted the relevant subset of individuals and computed a Euclidean distance on the raw repeat count data using the dist() function, without squaring the differences to retain the stepwise mutation model. We then applied AMOVA with the model specified as dmat∼pop, where ‘dmat’ represents the matrix of Euclidean distances and ‘pop’ the grouping variable. We extracted variance components with permutation testing (nperm = 1000) to generate *p* values for statistical significance, on whether the observed variance components (specifically the between-population variance) differ significantly from those expected under the null hypothesis of no genetic structure.

From the AMOVA output, the variance components between populations (σbetween) and within populations (σwithin) were extracted and pairwise *R*_ST_ distances were calculated using the formula:

$${Rst}=\frac{{{{\rm{\sigma }}}}{{{\rm{between}}}}}{{{{\rm{\sigma }}}}{{{\rm{between}}}}+{{{\rm{\sigma }}}}{{{\rm{within}}}}}$$

where the ratio quantifies the proportion of the total genetic variance due to differences between populations. In cases where total variance was zero or incomputable, *R*_ST_ was recorded as missing. The computed *R*_ST_ distance for each population pair was assigned symmetrically in a distance matrix for visualisation and interpretation (Fig. S6).

The fact that 51% of Deep Maniots belong to a specific Y-haplogroup subclade (J-L930), renders this population an extreme outlier in the broader comparative dataset (Fig. S6). Despite this remarkable founder effect, Deep Maniots display high within-population variance, likely resulting from high mutation rate of Y-STRs leading to high haplotype diversity. While AMOVA-derived *R*_ST_ can offer valuable insights on population differentiation under a stepwise mutation framework, the inherent variability and potential for inflated within-population variance in extreme outlier populations like the Deep Maniots can lead to potential bias and limit its interpretability. In particular, inflated within-group variance can dilute the between-population signal, potentially leading to biased *R*_ST_ values. Moreover, the assumption of the strict stepwise mutation model underlying *R*_ST_ estimation might not fully capture complexities arising in such extreme cases.

To assess genetic differentiation among populations, we employed an alternative approach, where we estimated pairwise genetic distances based on allele frequency data using the *adegenet* package (v2.1.10)^117^ in R (v4.3.2). Individual-level Y-STR data were first converted into a genind object using the df2genind() function, specifying haploid codominant markers and assigning individuals to populations via a population vector. The derived genind object was then transformed into a genpop object using function genind2genpop(), aggregating allele frequencies at the population level.

Pairwise genetic distances between populations were computed using the dist.genpop() function, setting method to Rogers’ genetic distance (option 4)^76,143^, providing a Euclidean distance metric based on allele frequencies defined as:

$${D}_{4\left(a,b\right)}=\left(\frac{1}{\nu }\right){\sum }_{k=1}^{\nu }\sqrt{\,\left(\frac{1}{2}\right){\sum }_{j=1}^{{m}^{k}}{\left(\,{p}_{a\,j}^{k}-{p}_{b\,j}^{k}\right)}^{2}}$$

where *D*_*4(*_*(a, b)* is the genetic distance between two populations, *m* is the number of loci, and *p*^*k*^_*aj*_ and *p*^*k*^_*bj*_ are the allele frequencies at locus *j* in populations a and b, respectively. This approach provides a robust approximation of genetic differentiation suitable for multivariate analyses such as MDS. The resulting distance matrix was converted to a full symmetric matrix for downstream visualisation and interpretation.

Rogers’ distances, by focusing on aggregated allele frequency differences, potentially provide a more robust and reliable measure of genetic divergence compared to *R*_ST_ distances, in this specific context. In particular, by summarising the overall allele frequency profile, this approach is less sensitive to the high variance within a dominant haplogroup and might provide a more robust estimation of genetic differentiation that reflects the cumulative discrepancies in allele frequencies across all loci without heavily penalising extreme within-population variability. This stability becomes especially advantageous when contrasting populations with varying internal diversities and underlying genetic structures, particularly in the presence of extreme outlier populations. This is particularly relevant in our case, given the Deep Maniot population’s outlier status. Given this we opted for the use of Rogers’ distances as our primary metric for inter-population comparisons.

### Dimensionality reduction and graphical representation of population distances

To visualise patterns of genetic similarity among populations, we applied NMDS analysis to the pairwise genetic distance matrix derived from Rogers’ distance (see above), using the metaMDS() function from the *vegan* package (v2.6-4) in R (v4.3.2)^119^. A two-dimensional solution (*k* = 2) was specified and we allowed up to 200 random starts (trymax = 200) to ensure convergence on a stable configuration. NMDS is a rank-based ordination method that represents the relative dissimilarities between populations in a reduced-dimensional space, preserving the order of distances rather than their absolute values. To enhance clarity in the ordination plot and minimise potential bias from populations with extremely high genetic distances or small sample sizes, specific populations (e.g. Georgian, Ossets_Iron, Kubachi, and Shapsug) were excluded from the final visualisation. The NMDS plot was generated using the *ggplot2* package^120^, providing a two-dimensional depiction of the genetic landscape across populations. The stress value, a measure of the mismatch between the original distance matrix and the ordination configuration, was used to assess the goodness-of-fit. Lower stress values indicate a better representation of the data in reduced dimensions, with values below 0.1 generally considered indicative of a good fit.

As an alternative to NMDS, we applied Uniform Manifold Approximation and Projection (UMAP) using the *umap* package (v0.2.10.0)^144^ in R (v4.3.2), to visualise genetic relationships among populations in a reduced-dimensional space. UMAP is a non-linear dimensionality reduction technique that preserves both local and global structure in high-dimensional data, making it well-suited for exploring complex genetic distance matrices, offering an alternative visualisation of genetic relationships. The analysis was applied directly to the pairwise genetic distance matrix derived from Rogers’ distance using the umap() function with default configuration settings (umap.defaults) to maintain consistency with common usage standards and specifying the input type as ‘dist’. The resulting two-dimensional layout was extracted and used to construct a scatterplot, with each point representing a population and labelled accordingly.

NMDS and UMAP represent different approaches of dimensionality reduction. UMAP is designed to preserve local neighbourhood structures. It models data as a high-dimensional graph and optimises a low-dimensional layout that retains connectivity within that graph, therefore clusters are easier to detect and interpret visually. However, UMAP usually does not preserve global distances or the overall shape of the original space, therefore, the spatial placement of outliers may understate their true genetic distances. NMDS optimises a configuration such that the rank order of pairwise distances is preserved. It prioritises getting the relative dissimilarity (i.e. which populations are genetically more distant from each other) correct, even at the expense of local spacing. As a result, genetically close populations may appear crowded in overlapping clusters, while outliers are usually accurately positioned as genetically distinct. Since Deep Maniots represent an outlier population, we opted for NMDS as the main method for the graphical representation of pairwise genetic distances. We should note that UMAP results in nearly identical plots with those generated by NMDS (Fig. S7).

### Mitochondrial haplogroup assignment

We obtained off-target mtDNA data from BigY-700 testing for 50 of the 71 NGS-tested individuals who also descended from Deep Maniot matrilines, while one additional mtDNA sequence was obtained by querying the FamilyTreeDNA’s database. Since these are off-target reads (compared to FamilyTreeDNA’s targeted mtDNA test), we can expect a slightly higher risk of NUMT reads being included in the analysis. However, these occur at such a low frequency compared to mtDNA that they could at most result in a heteroplasmy call, which would be excluded from the phylogenetic analysis. We use a threshold of 20% allele depth difference from the majority call for a site to be classified as heteroplasmic. Additionally, FamilyTreeDNA employs an mtDNA analysis pipeline similar to the Broad Institute’s best practices workflow for Mitochondrial short variant discovery, incorporating normalisation steps such as MergeBamAlignment --UNMAP_CONTAMINANT_READS to ensure data accuracy and integrity.

The resulting FASTA files were then uploaded into Haplogrep 3^145^, which utilised PhyloTree build 17^146^ to determine mitochondrial haplogroups and identify local private mutations. The phylogeographic affinities of Deep Maniot mtDNA haplogroups were established by comparison against FamilyTreeDNA’s mtFull database, which comprises >260,000 mitochondrial genomes, as well as all publicly available mtDNA sequences of the same macro-haplogroup on BLAST and GenBank.

### Median-joining network construction on mtDNA sequences

To explore the phylogenetic relationships between Maniot mitochondrial lineages and those of modern and ancient populations, we constructed median-joining networks (MJNs) for four major mitochondrial haplogroups observed in the Deep Maniot sample: HV119, H7c1k1, U1a1d1d, and U5a1b1. For modern samples, complete mitochondrial consensus sequences were retrieved directly from GenBank, using accession numbers reported in the literature (Supplementary Data 15). For ancient samples, consensus sequences were retrieved from GenBank and the Allen Ancient DNA Resource (AADR)^147^. In instances where ancient consensus sequences were not directly available, we obtained the corresponding raw sequence data (BAM files) from the European Nucleotide Archive (ENA). SAMtools (v1.18)^113^ was initially used to ensure that the BAM file is sorted and indexed and the consensus sequences were then reconstructed using ANGSD (v0.931)^148^ following a pipeline suitable for lower coverage or fragmented aDNA data, using options -doFasta 2 -doCounts 1 (chooses the most common base at each locus) -minQ 5-25 (sets the minimum base quality discarding bases with quality scores below a given threshold—increments of 5 in this case) -trim 5 (trims 5 bases at read termini to account for the fact that nucleotide misincorporations due to damage are common in aDNA)^149^.

Following the compilation of consensus sequences for each haplogroup, we aligned sequences using MEGA^150^ and constructed median-joining networks (MJNs) using PopArt v1.7^151^, for each haplogroup of interest. Sample nodes were annotated by population or geographic origin, using population metadata. These networks were used to assess the degree of haplotype sharing between Maniots and other populations, and to visualise potential phylogeographic structure within each haplogroup.

### qpAdm admixture modelling

One of the Deep Maniot lineages has been found in an ancient individual (I8216) from Roman Era Empuries in present-day Catalonia, Spain. This individual was previously modelled as having Aegean ancestry^152^. To model the autosomal ancestry of I8216, we employed *qpAdm* from ADMIXTOOLS v.2.0.0^153^ by using a base set of references approach^154^. A model was accepted as statistically plausible if its *p* value was ≥0.01, as followed by previous work. *Z*-scores were automatically estimated by our *qpAdm* script, and only values to close to or >*Z* = 3 are reported. An extensive description of our *qpAdm* models and the rationale behind the chosen source and reference populations can be found in the Supplementary Data.

## Supplementary information

Supplementary Information

Description of additional supplementary files.

Supplementary Data 1–15

## Data Availability

Following consultation with the Deep Maniot community, and in compliance with FamilyTreeDNAs privacy terms, specific sequencing data (BAM, VCF and FASTA files) are restricted and will not be deposited in a public repository. Applications for strict scientific use of the data can be made by a joint request to L.R.D., A.D.M., and A.H. Access will be granted upon consultation and approval by each individual participant who volunteered in this study, or their close family members, in cases where the volunteer is deceased.
