# Supplementary material for: Uniparental analysis of Deep Maniot Greeks reveals genetic continuity from the pre-Medieval era
Source: Commun Biol. 2026 Feb 4;9:157. doi: 10.1038/s42003-026-09597-9 (PMC12873217; doi:10.1038/s42003-026-09597-9)
Supplement: Supplementary file 1 — Supplementary Information [file 42003_2026_9597_MOESM1_ESM.pdf]

Supplementary Text 1.

1. Extended haplogroup discussion.

This section discusses the associations of all lower frequency haplogroups found in Deep Mani, which collectively account for approximately 30% of Deep Maniot patrilines (Table 1). As with J-L930, J-FTF87157 and R-FTE77744, most of these patrilines likely originate from the ancient Balkans and West Asia.

Haplogroup J-L26>J-PF5087>J-PF5160>J-Z387>J-FGC35461>J-Z39097>J-PH4244 (7% frequency in Deep Mani) has so far been found exclusively in the areas of Pyrgos Dirou-Areopolis and the northern border of Deep Mani (Fig. 4A). Although its sister branch is found in Poland (Fig. S3), most present-day testers under upstream lineage J-FGC35461 originate from West Asia, the Levant, and the Balkans, and one lineage that shares a TMRCA with J-PH4244 at ca. 2,500 BCE was also found in a Byzantine West Anatolian individual (Muğla 20187; Fig. 5)[1]. It is therefore likely that J-PH4244 arrived in Deep Mani during the Roman Period.

Some Deep Maniot lineages are characterised by related branches that have been found in Roman Period individuals from the Balkans who descended from intermarriage between local Balkan Iron Age groups and western Anatolians. These individuals carried Y-DNA lineages that included J-L26>J-PF5087>J-PF5160>J-Z387>J-Z40002>J-Z39993>J-Z39995>J-Z40011>J-Z39986>J-Z40003>J-FT106400* (2%; shared branch J-Z40002 with sample Muğla 20259 at ca. 2150 BCE)[1], J-L581>J-Z39726>J-Y3143>J-Z28329>J-Z39727>J-FT151551* (2%; same branch with individual 2041 from Croatia Sisak Pogorelec)[2] and G-L13>G-FT324622>G-FT275186>G-FTF91185 (6%; shared branch G-FT324622 with individual from Croatia Osijek)[3] (Fig. 5). Considering the dating and autosomal profile of these ancient samples, we suggest that the related lineages which are presently found exclusively in Deep Mani may originate from the same or a similar wave of Anatolian migration into the Balkans and other parts of the Roman Empire[3,4]. Indeed, parts of Deep Mani, especially Caenipolis, received immigrants from across the Greek-speaking world (including Cyprus) as well as more distant regions such as Judea[5].

Haplogroup J-PF7263>J-ZS4452>J-FT67172 (2% in Deep Mani) forms a clade with a present-day Sicilian, with a TMRCA estimated at 287 CE. The J-PF7263 branch has been found in an ancient individual (I8216) from the classical Greek colony of Empuries in Spain[6]. Although this ancient individual was interpreted by a previous study as having Aegean connections [6], our own autosomal analysis using *qpAdm* recovers significant North African ancestry for this sample and an association with Punic populations (see section on *qpAdm* below; Supplementary Data 8) who colonized southern Spain and North Africa[7]. The presence of related branch J-PF7263>J-ZS4452>J-ZS10589>J-ZS10568 in present-day Ashkenazi Jews also strengthens our suggestion that J-PF7263 as a whole is ultimately associated with the BA-IA Levant.

Other lineages with no close branches in the aDNA record but of likely West Asian-Levantine origins include J-L25>J-Z438>J-BY76232* (2%, related branches found almost exclusively in

Italy, with distant matches in Bulgaria and Iran, Supplementary Data 6) and J-L26>J-PF5087>J-PF5116>J-M67>J-L227>J-FT114604>J-Z44002>J-FTF77337 (1%; most basal branches of J-L227 are found in populations from the Caucasus, while related branch J-L227>J-Y165124 is found almost exclusively in Ashkenazi Jews[8]). Although related branches for these lineages contain present-day individuals from Western Europe, we suggest an arrival during the Roman period, when Eastern Mediterranean and especially Anatolian-related ancestry (including Y-chromosome haplogroups) became pervasive across the Roman Empire, even in the northernmost frontier regions[2–4,9].

The origins of haplogroup J-Z393>J-Z44288>J-Z44310>J-BY93436 (2%) are puzzling, due to the absence of relevant aDNA samples, and seemingly disparate present-day related matches in Armenia, Sudan, the Levant, and the Arabian Peninsula.

Two Deep Maniot haplogroups can be definitively associated with the BA-IA northwestern Balkans. Haplogroup E-V13 (E1b1b1a1b1a)>E-BY3880>E-Y16729>E-BY202063>E-FT64983 (1%) was found in a Roman Period individual from Trogir, Croatia (26702)[3], and haplogroup E-V13>E-BY3880 as a whole was particularly frequent and diverse in EIA Bulgaria[10,11]. Although up to 20% of present-day NGS-tested Greeks with E-V13 belong to subclades associated with the 10th and 14th century CE migrations of Aromanians and Albanians (Supplementary Data 9), none of them are found in E-BY202063>E-FT64983, despite extensive sampling in Albania (n = 1806)[12]. This suggests that E-BY202063>E-FT64983 arrived in Deep Mani prior to that period, most likely in Hellenistic or Roman times.

The same applies for two Deep Maniots who are found to be J-L283>J-Z600>J-Z631* (J2b2a1a1a1a1a1a, 2%). Haplogroup J-L283>J-Z600 experienced a major founder effect in the Bronze Age West Balkans, where it accounts for more than 70% of all patrilines[10]. The absence of any close matches with Albanians, Aromanians, or any other ethnic group where J-Z631 has been found indicates an introgression of this haplogroup in Deep Mani's distant past. Other lineages of J-L283>J-Z600 have been found in BA Greece[10,13,14], suggesting early contacts of the Greek world with the northwest Balkans.

Haplogroup R1a-Z93 (R1a1a1b2)>R-BY61693, which has been found in a single Deep Maniot individual (1%), ultimately originates in the MBA Sintashta and Srubnaya cultures of the Pontic-Caspian steppe[15], and was a characteristic lineage of later Indo-Iranian nomadic peoples such as the Sarmatians[16,17]. This lineage likely represents introgression into Deep Mani from the Dodecanese in the southeastern Aegean, as indicated by STR matching to an individual from Karpathos at ca. 1650 CE (Fig. 3), and two more individuals from Rhodos at lower STR levels.

2. *qpAdm* admixture modelling.

1.1. Ancestry modelling for Roman Period individual I8216 from Empuries, Catalonia, with Bronze Age, Iron Age and Roman period populations

To characterise the distal genetic makeup of I8216, we used *qpAdm*[18,19] to rotate among earlier populations from the Aegean (Greece_BA), the Levant (Lebanon_MBA), North Africa (North_Africa_CA_IA) and contemporaneous indigenous Iberian groups (Spain_Classical_Greek_Iberian). This approach aimed at capturing the likeliest sources for the ancestry of I8216, using populations from both the eastern and western Mediterranean.

The fixed references included Cameroon_SMA, Spain_IA, Morocco_EN.WGC, Israel_MLBA, Morocco_Iberomaurusian, ISR_Natufian_EpiP, RUS_West_Siberia_HG, Russia_Karelia_HG, WHG, TUR_Pinarbasi_EpiP, Russia_Boisman_MN.

The results of this analysis demonstrate that I8216 is characterised by 20-30% North African-related ancestry (Supplementary Data 8), suggesting that this individual did not exclusively descend from Aegean or Levantine populations, as suggested previously[6]. We should note that our model could not discriminate whether the remainder of the ancestry of I8216 originates from Aegean or Levantine Bronze Age populations (Supplementary Data 8). However, as I8216 is a low coverage genome (43602 SNPs, Supplementary Data 8), the discriminatory power of our analyses may be suboptimal.

The presence of North African ancestry in individual I8216 may indicate an origin from Punic groups, which previous studies have shown that they comprised a diverse tapestry of ancestries, originating from populations across the Mediterranean[7,20].

1.2. Ancestry modelling for Roman Period individual I8216 from Empuries, Catalonia, with Punic populations

Based on the findings mentioned above, we aimed to test whether individual I8216 derived his ancestry from Punic individuals. To this end, we rotated using Aegean Bronze Age (Greece_BA) and Punic (Italy_IA_Roman_Punic_Profile, Tunisia_Kerkouane_Punic) individuals. We also included roughly contemporaneous populations from Lebanon (Lebanon_Late_Antiquity), Levantine-shifted Balkan individuals (Balkans_Roman_Levant_o)[3], and North African-related proxies (North_Africa_CA_IA). The results indicate that I8216 derives most or all of his ancestry from Punic-associated individuals (Supplementary Data 8), in line with the findings of our previous models.

3. Inferences on settlement patterns in Deep Mani based on the phylogeography of haplogroups J-L930 and J-FTF87157.

The geographical distribution and branching patterns of the predominant Deep Maniot haplogroup, J-L930, may offer tentative insights into historical settlement dynamics within the region. All four branches of J-L930 are characterised by early-splitting lineages located in western Deep Mani, particularly in Oetylo, Xoumero, Niklianiko, and Katopangi – the latter containing the earliest diverging branch, J-FTE86410, dated to ca. 850 CE (Figs. 6A, S4). This clustering may reflect an origin in western Deep Mani for a substantial portion of the present-day population's paternal ancestry. Individuals with subclade J-FTG29984 (ca. 1170 CE) in western Deep Mani originate

from villages adjacent to the only immediately available mountain pass that connects the western to the eastern half of the Mani Peninsula (Fig. S4), which might explain the presence of this lineage on both sides of the peninsula.

A subclade that is found almost exclusively in eastern Deep Mani is J-L930>J-Y251116 (Figs. 4B, 6A). This subclade experiences most of its branching in eastern Deep Mani from the beginning of the 14th century onwards (1306 CE; Fig. S4), which may be correlated with a wave of settlement into the region.

A study of the branching patterns of the second most frequent Deep Maniot haplogroup, J-FTF87157, indicates that eastern Deep Mani may have also received paternal input from the southwest. Subclades downstream of J-FTF87157>J-FTF86959 (1190 CE) are found in Taenaron-Kritiri and Lageia, with oral traditions linking them to migrational waves from Vatheia and nearby mountain villages (S1 Text). Indeed, the deepest split within J-FTF87157, subclade J-FTG43178 (910 CE), is formed by two individuals from Vatheia and Caenouria Chora, potentially validating oral traditions that identify this area as a population source for southeastern Deep Mani.

These branching patterns are in broad agreement with archaeological data, which indicate that the earliest phases of church construction are to be found almost exclusively in western Deep Mani[21–23]. However, the settlement of eastern Deep Mani may have involved complex processes not fully captured by the results of our genetic analyses. Megalithic structures are also found in the eastern half other Mani Peninsula, indicating habitation in Late Antiquity and Early Medieval times[24]. However, our genetic analyses indicate that the present-day inhabitants of eastern Deep Mani paternally descend from later migrational waves within Deep Mani that took place between 1200–1300 CE. It is therefore possible that the region was abandoned at some point prior to the 13th century and later resettled by individuals from western Deep Mani and the mountain villages situated between east and west Deep Mani. We stress that these hypotheses remain provisional and should be interpreted with caution given the limited number of lineages analysed.

4. A review of the Deep Maniot clans examined in our study.

Although the patrilineal clan system is one of the defining features of Deep Maniot society[25–28], only a handful of academic researchers have recorded their oral traditions of kinship, descent, historical movement and geographical partition within the area of Mani. Most formal research that has so far been undertaken primarily concerns the most prominent and historically attested clans, such as the Nikliani[26,29,30] and the Kondostavli[31–34]. Furthermore, the seminal works of Alexakis[25] on the sociology and anthropology of the clan system, Saitas[24,35] on Deep Maniot sociology, archaeology, and settlement patterns, and Katsafados[36–38], on Deep Maniot epigraphy, demography and history, have never been repeated. This is a significant knowledge gap, as the massive exodus of Deep Maniots to other parts of Greece and abroad, and the drastic change of social organization of Deep Maniot society since the mid-20th century[25], have significantly impacted the transmission of Maniot oral histories to younger generations. Based on our extensive field observations in Deep Mani, the vast majority of Maniot oral history is safeguarded by individuals over 60 years of age.

It is likely that we are witnessing the last generation of individuals with a comprehensive, firsthand understanding of Deep Maniot oral history and traditions.

However, citizen historians have painstakingly recorded the oral traditions associated with the Deep Maniot clan system. These include, but are not limited to, the currently unmatched – yet often self-published – works of Kyriakos Kassis[39–45,] Ioannis Lekakos[46], and Anargyros Koutsilieris[47,48], among others. In the sections below, we provide, to our knowledge, the most comprehensive and up to date overview of the oral histories of the Deep Maniot clans that we included in our study. The core of this analysis is based on the abovementioned works, whose veracity we tested and supplemented by our discussions with representatives of each of the examined clans (who frequently provided previously unpublished information).

The Nikliani Clan

The Nikliani arguably are the most prominent and recognisable clan in Deep Mani – so much so that their name became synonymous with the clan institution itself[39,40], even being used to describe powerful Maniot families that had no genealogical affiliation with the said clan[25,49]. First attested in the 16th century[27,38,47,50], the Nikliani historically dominated an area known as the Niklianiko, in the vicinity of the villages of Kita and Nomia in southwest Deep Mani (Fig. 1A). Over time, the Nikliani expanded into other parts of Deep Mani, including the south and southeast, and split into several subclans, such as the Liani, Michalakiani, Koutrouliani, Tsingrosaouliani, Kandiriani and the Giannakomicheliani[39,40]. In this study, we tested members of the Koutrouliani. Michalakiani and Liani subclans.

Despite their prominence, the genetic origins of the Nikliani are particularly complex. Our analysis shows that different Nikliani subclans fall on separate branches of haplogroup J-L930>J-FTE86410, interspersed among lineages belonging to other clans (Fig. 6A). We discuss each tested subclan in detail in the sections below.

In our interviews with three men of the Nikliani: Koutrouliani subclan, each originating from three different villages separated from each other by a maximum distance of 7 km, we established that although surrounded by other clans and Nikliani subclans, the Koutrouliani maintained their stories of shared kinship. All three interviewees mentioned the same common ancestor, Koutroulis, who lived in the village of Vatheia, many centuries ago. These oral histories of kinship are supported genetically, as all three Koutrouliani men share the haplogroup J-L930>J-FTE86410>J-FTE89060>J-FTF91224. This lineage represents the second oldest known instance of a Deep Maniot clan founder in our dataset, with a TMRCA dated to 1392 CE (Fig. 8).

However, other tested Nikliani subclans, such as the Liani and Michalakiani, belong to downstream subclades of J-FTE89060: specifically, J-FTE89060>J-FTE90738* (TMRCA at 1270 CE) and its daughter lineage J-FTE89060>J-FTE90738>J-FTG31870 (1620 CE) (Fig. 8). This suggests that the broader Nikliani identity may have originated within subclade J-FTE89060 (1163 CE), encompassing both the Koutrouliani (J-FTF91224) and other Nikliani lineages (J-

FTE90738* and its descendants). Alternatively, these findings could reflect two distinct patrilineal lines – J-FTF91224 and J-FTE90738* – that independently adopted the Nikliani identity, potentially driven by sociopolitical dynamics. The latter hypothesis is reinforced by a newly recorded tradition of some members of the Koutrouliani, who state that Koutroulis predated the Nikliani, and only later did some of his descendants fuse with the latter. It is therefore possible that the putative founder of the Nikliani belonged to haplogroup J-FTE89060>J-FTE90738 (and its daughter lineages), which is consistent with the oral tradition of the clan that postulates an origin in the 13th century[39].

Further evidence for the above hypothesis may stem from two additional clans – M1 from Alika, and M2 from Dri, both of which belong to subclade J-FTE89060>J-FTE90738*. Clan M1 is found in Alika, and their oral history suggests an origin from Nomia. Clan M2 has traditionally inhabited the villages of Dri and Keria and claimed an origin from Kita many centuries ago. Although Dri was inhabited by the Kondostavli clan in 1514[38], this was no longer the case by 1618, suggesting a possible population turnover, possibly triggered by the arrival of clan M2 into the village. Some, but not all of the clan elders pertaining to clans M1 and M2 state that "today we are distinct families, but our grandfathers said that in the past we were powerful Nikliani".

Regarding the mythological origins of the clan, it is often claimed that Nikliani are either of Greek origin[26,47,49] or that they descend from a Frankish or mixed Greek-Frankish lineage[47,50]. Some authors go as far as identifying Niklos with the Frankish Lord Nicolas II of Saint De Omer[30,39]. Given that all Nikliani belong to J-L930, a Frankish or non-Maniot origin is unsupported.

The Kosmades clan

(i) The Taenarian Kosmades

The Kosmades clan of Taenaron settled the southernmost areas of Deep Mani, from their alleged cradle at the village of Vatheia.

They are one of the earliest attested clans in Deep Mani[41], first appearing in written records in ca.1514 in the region of Lagia and Vatheia[38], and later in 1570 when a certain Kosmas appears as a signatory of a petition to the Doge of Venice[47]. Kosmades are believed to descend from locals living in the area since at least the Byzantine times[41]. They comprise two main subclans, the Gerandoniani and the Karabatiani, each supposedly descending from one of two brothers with an ultimate origin in their cradle of Vatheia, many centuries ago[39]. We confirm that both subclans of the Taenarian Kosmades share patrilineal descent at ca. 1422 CE, under haplogroup J-FTF87157>J-FTF86959>J-FTF89604.

(ii) The Kosmades of Katopangi

The other branch of the Kosmades is situated in settlements in the tribal area of western Deep Mani, known as Katopangi. The settlements of this branch of the Kosmades are not geographically contiguous with the Taenarian Kosmades, separated by ca. 15 km from each other.

They are first attested in written documents in the 18th century[39] and claim descent from a man known as Tsoulos from the village of Pangia in Katopangi[39–41]. As a result, the Kosmades of Katopangi are known as the Tsouliani Karabatiani, whose descendants we included in our study (n = 3), and found to belong to haplogroup J-L930 (J-FTE86410>J-FTE89060>J-FTE90738>J-FTF3280, TMRCA at 1620 CE; Fig. 8) – a different lineage to the Taenarian Kosmades.

It is important to mention that in our interviews, members of the Tagarouliani subclan of the Tsouliani mentioned an oral tradition that reported shared patrilineal kinship with the Nikliani, with their entire lineage ultimately originating from Constantinople – an origin story not uncommon among Deep Maniot clans[25]. It is possible that the alleged origin from the Nikliani is confirmed by the genetic data that we recovered, as all the Nikliani in our dataset are downstream of J-FTE89060, with most being at, or further downstream of lineage J-FTE90738 (Fig. 5A).

Based on the genetic findings presented in the main text of our study, we suggest that the kinship of the Kosmades of Katopangi with the Kosmades of Taenaron may have been the result of a political or military alliance called *"syndrofia"* (literally translated as companionship)[52], which over time transformed into a sense of blood kinship, a phenomenon which is not uncommon in Deep Mani's genealogical traditions[53].

The Kondostavli clan

The Deep Maniot clan of Kondostavli were a large and powerful clan that was extensively documented in the historical record at least since 1514[38]. The Kondostavli in the present-day are subdivided into two subclans, each occupying two discrete areas of Deep Mani separated from each other by ca. 11 km. These two subclans comprise the Kondostavli of Kounos (also called Gerakariani) and the Kondostavli of Xoumero[39]. It is likely that a third and less well-known branch still exists in the Northeast of Mani at the vicinity of the Karyoupoli and Vachos villages. This is based on historical evidence of their presence there[56] in combination with oral traditions of families living in the Northeast of Mani in the present day[57], which we also recorded in our interviews with Kondostavli families. All the Kondostavli clan members whom we interviewed unanimously identify the area of Xoumero as the cradle of the surviving members of the clan. Some clan members may have also migrated to the island of Zakynthos and Italy[54].

By sampling both the Gerakariani (n = 1) and the Xoumero Kondostavli (n = 2), we confirm their tradition of shared descent, as all testers belong to subclade J-L930>J-FTA23105>J-FTG28332>J-FTG42117, which dates to ca. 1479 CE (Fig. 8) – a chronology that is remarkably close to the first attestation of the clan in 1514. Remarkably, the Gerakariani, who are mentioned as a prominent family of Kounos at least since 1571[33,34], have always maintained their oral traditions of kinship to the Xoumero Kondostavli despite being surrounded by occasionally hostile clans, the

Tagarouliani. Furthermore, the subclade of the Kondostavli of Kounos, J-FTG42117*, is found nowhere else in Katopangi, based on our dataset.

When we interviewed members of the Gerakariani branch of the Kondostavli clan, we were informed that they settled in the village of Kounos sometime between the 15th-16th centuries CE, originating from the village of Tsopakas (1.6 km from Dryalos). This tradition is in agreement with historical records, which demonstrate that Kounos was exclusively inhabited by the Kondostavli in 1514[38], and matches the TMRCA of their haplogroup subclade (1479 CE). In their battles against the Tagarouliani, we were told that the Gerakariani allegedly recruited both Kondostavli (from other villages), as well as displaced clans from Erimos (in Xoumero), Briki and Stavri (Katopangi), demonstrating the presence of a network of well-established strategic alliances in the region.

In our interviews with two Kondostavli families from Xoumero, we were informed that both originated from a certain Kondostavlos, who settled in the village of Dryalos between 1500-1600 CE. Indeed, both families form a subclade, J-L930>J-FTA23105>J-FTG28332>J-FTG42117>J-FGC42527, with a TMRCA at 1539 CE, confirming both their oral tradition of kinship, and the chronology of the alleged settlement of Dryalos. In the Ottoman census of 1514, however, different families inhabited Dryalos; this suggests that the Kondostavli of Xoumero settled and replaced the previous residents of the village sometime after the census was taken.Oral tradition often assigns a Byzantine noble origin to the Kondostavli[41,55] and different authors specifically claim they came to Mani from Constantinople[31,55]. Given that all Kondostavli descend from Deep Maniot-specific lineage J-L930, an origin from Constantinople is not supported.

Other examined clans

In the section below we provide an overview of the other clans that we included in our analysis.

<u>Clans with alleged descent from Byzantine nobility</u>

Komnini-Stefanopouli: This is one of the best-known Maniot clan due to their thoroughly documented emigration from Mani to Corsica in 1676[55,58].

They were first recorded in 1576 in a petition to the Venetians[34] and again in 1612 in a letter from Theodoros Stefanopoulos to a relative in Zante[59].

The clan was situated around the vicinity of Oetylo village (slightly further north of the boundaries of Deep Mani; Fig. 1A) and alleged descendants, who did not emigrate to Corsica, are still situated in the same area subdivided in numerous subclans, such as the individual that we tested.

This clan has one of the most interesting and prestigious origin myths as they claim descent from the last Emperor of the Byzantine succession state of Trebizond, David Komninos through his son Nikiforos[60,61]. Some members of the clan even suggested an affinity to the Medici family of Italy. Considering that the members of this clan descend from lineage J-L930>J-Y251116*, a haplogroup exclusive to Mani, an origin from the Komnini dynasty (who originated from Thrace) is unlikely. We should note that some members of the Kalkandiani clan claim a possibly affinity

to the Komnini. Given that the Kalkandiani clan are characterized by lineage J-Y251116> J-FTD87562*, a close relationship to the Komnini is unlikely.

Fokades: Another prominent Maniot clan of alleged Byzantine descent is the Fokades clan[55].

The clan has been historically located around the northeast border of Deep Mani, especially in the villages of Karyoupolis and Vachos.

Members of the clan claim to be part of the nobility that settled in Crete after the recapture of the island by Nikiforos Fokas in 961 CE and eventually made their way to Mani after the Ottoman conquest of the island around 1650 CE[43]. However, they are first recorded in Mani at least since 1571, when a Kalapothos Fokas was a signatory of a letter from the Maniots to the Doge of Venice[33].

The Fokas' lineage pertains to haplogroup J-L581>J-Z39726>J-Y3143>J-Z28329>J-Z39727>J-FT151551*. Although upstream branch J-Z39727 has been found in Cretans[62], the TMRCA with J-FT151551* is distant (1500 BCE). We consider a recent (within the last 1,000 years) origin from Crete as unlikely, unless there is significant unsampled diversity of J-Z39727 in Crete, which would be responsible for the deep splits within this haplogroup among Greek populations.

Clan X: Clan X (anonymised as requested by the clan) was a powerful clan comprising 8-9 families that settled the villages of Kotronas, Skoutari, Riganohora and Alepou in northeast Deep Mani. According to oral tradition, they originate from the Alika-Kyparissos villages, 35 km to the south, many centuries ago[25,39]. One sequenced member of clan X was found to belong to haplogroup G-FT275186>G-FTF91185>G-FTG30284, which forms a subclade with another tested individual from Alika at 1284 CE, supporting the oral traditions of an origin from Alika. However, the precise timeframe in which clan X's ancestors left Alika cannot presently be established. We also discovered an additional, SNP-tested member of clan X in the autosomal database, who was found to pertain to G-Z29424, upstream of G-FTF91185>G-FTG30284, and almost certainly belonging to the latter lineage. Haplogroup G-FTF91185 is found in moderate frequencies in Deep Mani (6%), where it is extremely localised, observed in three specific areas, namely Alika-Kyparissos, Pachianika, and Kotronas (Supplementary Data 1), with the middle region being equidistant to the other two (ca. 17 km). This suggests that the matching between clan X and the individual from Alika is unlikely to be attributed to chance.

Korogones:

The Korogones is a less well-known Deep Maniot clan yet one which appears to have been prominent in the 16th and 17th centuries. The family name first appears in the 1514 Ottoman census (TD80), where they are listed among the most numerous and powerful families in Apano Boularii (their cradle), Karynia, and Pangia, with smaller numbers in Ochia and Kounos[38]. They are also mentioned in 1576, when a priest called Father Korogonas signed a petition to release a Maniot condemned to death[34]. In the village of Ochia, although the Korogones were a minority in 1514[38], they became the dominant family by 1618, when the village was listed as Ozia di Chorogon in the

Venetian census[27]. Although the Korogones were historically settled around in western Deep Mani, they also expanded and founded[27] the eponymous village of Korogonianika in southeastern Deep Mani, sometime prior to 1618, when the village was first mentioned in the Venetian census[27]. Today, the Korogones are among the rarest families in Deep Mani, known almost exclusively from their southeastern outpost of Korogonianika, while Ochia is currently inhabited by the Smailiani clan (S1 Text), whose members are not aware of any kinship with the Korogones.

Our genetic analysis shows that the Korogones are the earliest recovered clan founders in Deep Mani, with genetic origins traced to 1350 CE (Fig. 8). Remarkably, sequenced (n = 1) and STR-tested (n = 2) Smailiani men from Ochia form a clade with a Korogones clan member from the village of Korogonianika (subclade J-L930>J-FTA23105>J-FTA23355) (Fig. 6A), despite their geographical separation. This suggests that Ochia, as indicated by both Ottoman and Venetian censuses, was originally a Korogones settlement. Its inhabitants later adopted a different clan identity after the 17th century; clan fission and splitting were not uncommon in Deep Mani[25,39,40].

We propose that the observed genetic relationship between men from Ochia and Korogonianika may stem either from the original Korogones population documented in Ochia in 1618, or from slightly later Korogones settlers originating from Boularii. Indeed, in the 17th century, the Nikliani clan expanded into Boularii[38], which likely exerted pressure on Korogones communities and contributed to their geographical fragmentation and decline.

According to Kassis, the oral tradition of the family alleges an origin from Mystra around 1600[41]. This assertion was repeated in our interviews with members of the Korogones clan from Korogonianika. However, because the Korogones belong to a Deep Maniot-specific subclade of J-L930 and are documented in 16th-century censuses, a 17th-century origin from Mystra is unlikely.

Clans of alleged descent from medieval soldiers

Hourigianei: This is a clan settled around the broader area of Pyrgos Dirou and Aeropolis. It is claimed that they originate from a relative of the Greek revolutionary Ypsilantis with ancestry from Pontos[44]. The same author later claimed that the progenitor was a Maniot who returned to his homeland from other parts of the Byzantine Empire after the fall of Constantinople to the Ottomans[39].

Nikandriani: The Nikandriani clan also settled in the Pyrgos Dirou area, following the earlier settlement of the Hourigianei clan[44]. According to Kassis, the clan descends from an Anatolian soldier named Nikandros, who was part of an army sent by Emperor Michael VII Palaiologos in 1262 to defend the Romans against Slavic incursions in southern Peloponnese[44]. Several contemporary families in nearby villages claim descent from Nikandros. The Nikandriani clan comprises the Michaliani, Skandaliani, and Georgiliani subclans, with the Georgiliani further branching into the Grigoriani and Pavliani clans.

The tested Nikandriani, like the abovementioned Hourigianei, belong to haplogroup J-Z39097>J-PH4244>J-FTF91234, with a TMRCA at 1072 CE, which is close to the alleged origin of the clan

from Anatolia, in 1262. However, considering that these chronologies might well be coincidental, and the absence of present-day testers from Anatolia within this clade, we treat the veracity of this oral history as inconclusive.

Clan L: This is one of the major clans of the Kotronas area in the Northeast of Deep Mani[39]. They first appear in written records in the 1715 calendar of the practical doctor Papadakis. It has been claimed that their progenitor was a Byzantine of Albanian origin[46], or a non-Maniot Greek stradioti[39]. Given that this clan belongs to J-L930, which is so far exclusive in Deep Mani, an origin from Albania or outside the Mani Peninsula is unsupported.

Clans of alleged foreign descent

Aravouhii: The Aravouhii clan have historically settled around the villages of Vatheia and Tsikalia[40].

They first appear in the 1514 Ottoman census, inhabiting the villages of Porachia, Vatheia, and Kalonii[38]. They are next mentioned in 1571, in a letter to the Doge of Venice where Aravouseos Ioannis from Vatheia is a co-signatory[33].

There are several origin myths regarding this clan. One such myth claims that they were traders originating from the Sicilian town of Ragousa or alternatively the Adriatic city of Ragusa (modern day Dubrovnik in Croatia) and that they were a lesser clan "submitted" to the stronger Kosmades clan[41]. However, some studies mention an oral history stating that they were North African slaves brought to Mani by pirates[42] or Arab settlers[39]. Given that the single tested member of the Aravouhii belongs to lineage J-FTF87157>J-FTF86959>J-FTF89604, which originates from the Greek Bronze Age and is found in the Taenarian Kosmades, a foreign origin is unsupported. It is possible that the Aravouhii were originally part of the Kosmades clan (also defined by J-FTF89604), but by 1514 they had become a separate clan – the reverse scenario is equally plausible. However, more branches of the Aravouhii need to be tested before definitive conclusions can be drawn.

Giannitsariani: The Giannitsariani clan has settled in the village of Elia, in the Katopangi area of Deep Mani. One origin story states that they descend from an Ottoman janissary who fled to Mani[41] possibly after a coup in Constantinople in the 1600's[40]. This origin myth was also repeated during our interviews by members of the clan. Another version of this origin myth claims that the said janissary was of Greek Maniot origin to start with[41]. We consider the latter scenario as unlikely, as the presence of Ottomans in Mani was very limited[27,47], and there are no records, to our knowledge, of Mani serving as a source of janissaries. Given that the Giannitsariani belong to haplogroup J-L930, a foreign origin is unsupported.

The clan suggests that their ancestors first settled Briki, and due to clan warfare, they were forced to migrate to the village of Kita around 1700 CE. The Giannitsariani were soon expelled from Kita following conflicts with the preceding Kostarakos clan. After joining forces with neighboring Kondostavli clan members and the more distant Nikandriani of Xoumero, they founded the village

of Elia. Members of the clan occasionally migrated to other villages, such as Agia Kiriaki and Dri, where they founded distinct subclans.

Smailiani: This is a clan settled exclusively in the village of Ochia, today comprising 15-20 families. It is said that at least some of the families moved to Ochia from Boularii in the 1700s, after a feud[39].

It has been claimed that they have North African descent from a progenitor called Ishmael[39]. In our interviews, we recorded an alternative origin story which claims that the Smailiani were Deep Maniots that were captured by pirates and taken to North Africa as slaves. At least some were able to buy their freedom return to Mani after 20-30 years in captivity.

Given that the Smailiani belong to J-L930, a foreign origin is unsupported.

<u>Other examined clans:</u>

Aravandiani: Aravandiani is an old clan of the Pyrgos Dirou area which allegedly preceded the Nikandriani clan in the region. They currently comprise 4-5 families[44].

Xyphomachaeridiani: The members of this clan claim that they were the first to settle the village of Lageia. The clan currently comprises 7-8 families[39]. Given that the Xyphomachaeridiani tester belongs to haplogroup J-Z28547>J-Z467>J-Z447>J-L210>J-Y4034>J-Z44002>J-FTF77337, which is so far unique in Deep Mani, the oral history of the clan, which claims the absence of patrilineal kinship with the other clans of Lageia, is confirmed. The first Ottoman census (1514) mentions the Machaeris and Skyphos families from Lageia, whose members may have merged to form the progenitors of the Xyphomachaeridiani[38], assuming that the original name was Skyphomachaeridiani.

Pilokokiani: This is one of the largest clans in Lageia. Their oral traditions state that they were the second settlers of the village (after the Xyphomachaeridiani). Before settling Lagia they allegedly inhabited mountainous areas of Deep Mani[39]. There is a currently unverified claim of a possible connection with the Giatriani clan[43].

Ritsiani: The Ritsiani are another large clan of Lageia and adjacent Spira[48]. Similarly to the Pilokokiani, they claim to have been among the earliest arrivals in the region[39].

Mavrokokiani: Another clan with purported ancestral connections with the first settlers of Lageia[39].

Given that the Pilokokiani, Ritsiani, and Mavrokokiani belong to lineages at or downstream of J-FTF87157>J-FTF86959>J-MF220902>J-Y297479 (TMRCA at 1415 CE), they all descend from a single ancestor who likely colonized Lageia in the 15th century, if not earlier.

Clan G. Members of clan G occupy both the village of Lageia[63], and the mountainous area around Leondaki – their alleged village of origin[39]. Some authors consider all members of clan G as a subclan of the Pilokokiani[48], whereas others claim that clan G was merely allied to the latter and

not related by blood[39]. Another version of their origin story has them as a subclan of the Kourikiani clan of Kita village[39].

We confirm that clan G is unrelated to the Pilokokiani, as the former belong to lineage J-L930>J-FTE86410>J-FTE89060>J-FTE90738>J-FTF91250 and share a TMRCA at 1415 CE with a man from Moundanistika – a village close to Leondaki.

Papadiani: This is one of the largest clans in Nyfi village on the eastern side of Deep Mani. According to the oral tradition of the clan, whose members we interviewed, they settled Nyfi around the early 1800s from the village of Alika. The clan comprises around 7-8 families.

Rozakides: This is an historical clan of Nyfi village which claims descent from the notorious pirate Rozos. They are possibly the first settlers of Nyfi village according to the oral tradition. A certain Roseas-Rozeas was mentioned as a native of Taenaron, who served as the skipper for the scholar Cyriac of Ancona, who visited Deep Mani in the 15th century[64]. Given that Taenaron used to refer to the area of Alika-Kyparissos, it is possible that the Roseas mentioned by Cyriac of Ancona may have been a member of the Rozakides clan. However, we cannot be certain that the Rosea in question was not allied with the contemporary Rousseas family of Dri, a village that was visited by Cyriac of Ancona.

Lagonikiani: In our interviews with a member of the Lagonikiani clan, we were told that the founder of the clan settled Nyfi from Alyka in the 19th century, due to a clan feud.

The Papadiani, Rozakides, and Lagonikiani clans belong to haplogroup R-Z2106>R-FTE77744>R-FTE77876, a haplogroup that has so far been found exclusively in Alika, Nyfi, and Xoumero. Given that these clans settled Nyfi in the last few centuries, the two main areas of origin of this haplogroup are to be found in Alika and Xoumero, which are separated from each other by 13 km.

Felouriani: The Felouriani clan, originating in the broader Kotronas area, was one of the largest in size and economic power in the region, from which many other families descended. According to a version of their origin story, the family originated from Molos in Lamia, where they were active as anti-Ottoman insurgents. After a conflict with the Turks, they fled to the Peloponnese, initially settling in Agriani. They later relocated to Vatas in Mani[65]. Their feud with clan L in the area is well documented[39].

Katrabachiani: The Katrabachiani are listed among the clans that inhabited the village of Kounos. Kassis mentions them as being related to the Kosmades of Katopangi[40], although this may be erroneous. In our interview with members of the Katrabachiani clan, we were told that the clan is not closely related to any other clan from Kounos or elsewhere and that a subset of their clan founded the nearby settlement of Karavas in 1855. One of the numerous subclan names of the Katrabachiani is mentioned in a nearby church inscription dated to 1265–1270 CE[37]. This family name is exclusively associated with the region of Kounos, within the Katrabachiani clan. If there is a biological connection between the 13th century individual and present-day Katrabachiani

(which remains to be demonstrated), then this might represent the oldest recorded family name that still survives in Deep Mani. Remarkably, the tested Katrabachiani individual is found to belong to haplogroup J-L930>J-FTA23105>J-FTG28332*, with a TMRCA at 1043 CE. Given that downstream branches include the Kondostavli (whose origin lies in Xoumero-Katopangi), a longstanding presence of the Katrabachiani in the area of Xoumero-Katopangi is a parsimonious interpretation.

Kaouriani: The members of this clan traditionally inhabited villages of Katopangi, although all claim an ultimate origin to the village of Kita (Supplementary Data 1). The Kaouriani were among the most powerful clans of Mani and some of its members participated in the last large-scale blood feud of Deep Mani, which shook the entire region in the 19th century[39–41]. Two Kaouriani testers belong to lineage J-L930>J-FTE86410>J-FTE89060>J-FTE90738>J-FTG8757, and share a common ancestor at ca. 1459 CE, rendering them among the oldest clans in our dataset (Fig. 8).

Clan S: Members of this clan claim to be allied, or even "share the same blood" with the abovementioned Kaouriani clan. Although traditionally listed among the powerful families of Katopangi, clan S has become almost extinct in Deep Mani over the last 200 years, as most of its male members were killed in clan warfare, or emigrated out of the region[39,40]. Members of clan S have always stated that they were among the first settlers of the Katopangi region. We report that the single tested member of clan S in our dataset belongs to Y-DNA haplogroup J-Z393>J-Z44288>J-Z44310>J-BY93436* – an exceptionally rare haplogroup on a global scale, with no close matches since 6,500 BCE[66]. Clan S shares no patrilineal kinship with any other family in the Katopangi area, where J-L930 is found at ca. 90% frequency (Fig. 4A; Supplementary Data 2), or Deep Mani more broadly. As a result, the purported relationship of clan S with the Kaouriani is likely the result of allyship (*syndrofia*), where unrelated clans fuse and form a new clan for economic and strategic reasons.

References

1. Lazaridis, I. *et al.* A genetic probe into the ancient and medieval history of Southern Europe and West Asia. *Science (1979)* **377**, 940–951 (2022).
2. Antonio, M. L. *et al.* Stable population structure in Europe since the Iron Age, despite high mobility. *Elife* **13**, e79714 (2024).
3. Olalde, I. *et al.* A genetic history of the Balkans from Roman frontier to Slavic migrations. *Cell* **186**, 5472-5485.e9 (2023).
4. Antonio, M. L. *et al.* Ancient Rome: A genetic crossroads of Europe and the Mediterranean. *Science (1979)* **366**, 708–714 (2019).
5. Gardner, C. A. M. The Mani Peninsula in antiquity: an archaeological, historical, and epigraphic investigation into regional identity. (The University of British Columbia, Vancouver, 2018).
6. Olalde, I. *et al.* The genomic history of the Iberian Peninsula over the past 8000 years. *Science (1979)* **363**, 1230–1234 (2019).
7. Ringbauer, H. *et al.* Punic people were genetically diverse with almost no Levantine ancestors. *Nature* https://doi.org/10.1038/s41586-025-08913-3 (2025) doi:10.1038/s41586-025-08913-3.
8. Waldman, S. *et al.* Genome-wide data from medieval German Jews show that the Ashkenazi founder event pre-dated the 14th century. *Cell* **185**, 4703-4716.e16 (2022).
9. Gretzinger, J. *et al.* Ancient DNA connects large-scale migration with the spread of Slavs. *Nature* https://doi.org/10.1038/s41586-025-09437-6 (2025) doi:10.1038/s41586-025-09437-6.
10. Davranoglou, L.-R., Aristodemou, A., Wesolowski, D. & Heraclides, A. Ancient DNA reveals the origins of the Albanians. *bioRxiv* 2023.06.05.543790 (2023) doi:10.1101/2023.06.05.543790.
11. Lazaridis, I. *et al.* The genetic history of the Southern Arc: A bridge between West Asia and Europe. *Science (1979)* **377**, eabm4247 (2022).
12. Alban Lauka; Gjergj Bojaxhi. RRËNJËT. Projekti Shqiptar i ADN-së. *rrenjet.com* https://rrenjet.com/ (2023).

13. Yediay, F. E. *et al.* Ancient genomics support deep divergence between Eastern and Western Mediterranean Indo-European languages. *bioRxiv* 2024.12.02.626332 (2024) doi:10.1101/2024.12.02.626332.

14. Skourtanioti, E. *et al.* Ancient DNA reveals admixture history and endogamy in the prehistoric Aegean. *Nat Ecol Evol* **7**, 290–303 (2023).

15. Narasimhan, V. M. *et al.* The formation of human populations in South and Central Asia. *Science* **365**, (2019).

16. Gnecchi-Ruscone, G. A. *et al.* Ancient genomic time transect from the Central Asian Steppe unravels the history of the Scythians. *Sci Adv* **7**, eabe4414 (2021).

17. Schütz, O. *et al.* Unveiling the Origins and Genetic Makeup of the 'Forgotten People': A Study of the Sarmatian-Period Population in the Carpathian Basin. *bioRxiv* 2024.10.04.616652 (2024) doi:10.1101/2024.10.04.616652.

18. Patterson, N. *et al.* Ancient Admixture in Human History. *Genetics* **192**, 1065–1093 (2012).

19. Harney, É., Patterson, N., Reich, D. & Wakeley, J. Assessing the performance of qpAdm: a statistical tool for studying population admixture. *Genetics* **217**, (2021).

20. Moots, H. M. *et al.* A genetic history of continuity and mobility in the Iron Age central Mediterranean. *Nat Ecol Evol* **7**, 1515–1524 (2023).

21. Seifried, R. M. The Legacy of Byzantine Christianity in the Southern Mani Peninsula, Greece, after Imperial Collapse. in *Rituals, Collapse, and Radical Transformation in Archaic States* 56–76 (Routledge, New York, 2020). doi:10.4324/9780429278020-4.

22. Seifried, R. M. & Gardner, C. A. M. Maintaining Distinction: Local Identity in the Remote Mani Peninsula, Greece, in the Classical, Byzantine, and Ottoman Periods. in *Joint Annual Meeting of the Archaeological Institute of America and the Society for Classical Studies* (New Orleans).

23. Mexia, A. Byzantine temple construction in the Peloponnese. The case of mid-Byzantine temples of Deep Mani. (National and Kapodistrian University of Athens, 2011).

24. Saitas, Y. *Megalithic in the Mani. Southern Peloponnese. A Contribution to the Settlement and Building Documentation. Part A. Digital Publication.* (2024).

25. Alexakis, E. P. The clans and family in the traditional society of Mani. (University of Ioannina, 1980).

26. Vagiakakos, D. Contribution on the Nikli-Nikliani of Mani. *Athina* **53**, 147–149 (1949).

27. Komis, K. *Population and Settlements of Mani. 15th-19th Centuries.* (University of Ioannina, Ioannina, 2005).

28. Komis, K. *Venetian Censuses of Mani-Bardounia(Early 18th Century) . Evidence of Economy and Historical Demography.* (Greek Letters, Athens, 1998).

29. Anagnostopoulos, G. *Laconian Chronicles. Amyclae, Amykli, or Nikli. Amykliani or Nikliani.* (Ellas, Athens, 1930).

30. Kougeas, S. Herkunft und Bedentung von neugriechisch Nikliani und famegii. *Glotta* **1**, 86–104 (1907).

31. Vagiakakos, D. Byzantine names and surnames from Mani. *Peloponnesiaka* **3–4**, 185–221 (1958).

32. Vagiakakos, D. The Melissini and Kontosavli of Mani in Zakynthos. *Yerabook of the Academy of Athens' Medieval Archive* **3**, 159–160 (1951).

33. Dokou, C. Revolutionary movements in Mani prior to the Battle of Lepanto and the opposition of Venie and Spain (1570-1571). *Laconian Studies* **1**, 212–266 (1972).

34. Tsiknakis, K. A case of blackmail of Venice by the inhabitants of Mani. (1575-1576). *Laconian Studies* **10**, 228–241 (1990).

35. Saitas, Y. *Mani*. (Melissa, Athens, 1992).

36. Katsafados, P. St. *The Castles of Maina. A Historical-Geographical and Archaeological Study.* (Self Published., Athens, 1992).

37. Katsafados, P. St. New Evidence on the dedicatory inscriptions (13th century) in the Church of Hagioi Theodoroi, Ano Poula, Inner Mani. *Bulletin of the Christian Archaeological Society* **36**, 275–288 (2015).

38. Katsafados, P. St. *Re-Imagining the Mani: Elegy to a Rural Population (11th to 17th c.). Volume II. The Megali Mani in the Ottoman Tapu Tahrir Defterleri The TD80 with Notes from the TD367, TD390, TD565, TD603 and TD677 (in Addendum).* (Paragraphos., Athens, 2025).

39. Kassis, K. *Genealogy or the Clans of Deep Mani.* (Self Published., Athens, 1978).

40. Kassis, K. *Lament Songs of Deep Mani. First Volume. Unpublished Texts and the Unknown Histories of Lament Songs and Families.* (Self Published., Athens, 1979).

41. Kassis, K. *Flowers of the Rock. Pre-Medieval, Medieval, and More Recent Families and Churches in Mani.* (Ichor, Athens, 1990).

42. Kassis, K. *The History of Mani*. (Self Published, Athens, 1977).

43. Kassis, K. *Lament Songs of Deep Mani. Second Volume. Unpublished Texts and the Unknown Histories of Lament Songs and Families.* (Self Published, Athens, 1980).

44. Kassis, K. *Anecdotes of Real People of Mani.* (Ichor, Athens, 1986).

45. Kassis, K. *The Krialis (Krialakos) Family of Mesa Mani.* (Self Published, Athens, 2007).

46. Lekakos, I. *Mani. Collections of History and Laography.* (Mani, 2004).

47. Koutsilieris, A. *History of Mani. Ancient-Medieval-Modern.* (Papadimas, Athens, 1996).

48. Koutsilieris, A. *Maniot Studies.* (Self Published, Athens, 1978).

49. Alexandrakos, D. *The History of Mani*. (Palamidis, Athens, 1892).

50. Dimitrakos-Mesisklis, D. *The Niklianoi.* (Athens, 1949).

51. Papadopoulos, T. Maniot settlers in Italy in the 17th century. *Laconian Studies* **6**, 182–258 (1982).

52. Vagiakakos, D. Linguistic elements of the Chronicle of Morea and the impact of the institutions of Frankish rule on the dialect and customs of Mani. *Peloponnesiaka* **16**, 465–512 (1985).

53. Andromedas, J. N. Maniat folk culture and the ethnic mosaic in the southeast Peloponnese. *Ann N Y Acad Sci* **268**, 199–206 (1976).

54. Vagiakakos, D. Mani and Zakynthos. in *Proceedings of the Third Panionian Conference (1965).* 17–32 (1967).

55. Kalonaros, P. *Mani - Castle of Tradition. Nature, History, Customs, Legends.* (Patsilinakos, Athens, 1981).

56. Etzeoglou, R. Karyoupolis. An abandoned byzantine city. *Laconian Studies* **9**, 3–60 (1988).

57. Patrikounakos, S. *Vachos Manis.* (1999).

58. Nicholas, N. A history of the Greek colony of Corsica. *Journal of the Hellenic Diaspora* **31**, 33–78 (2005).

59. Mertzios, K. Mani in the archives of Venice (1611-1674). *Laconian Studies* **1**, 83–173 (1972).

60. Cambiagi, G. *History of the Kingdom of Corsica Written by the Florentine Abbot Giovacchino Cambiagi Enriched with Dissertations, Documents, Papal Bulls, Annotations Etc. Volume XII.* (Scritta Dall'Abate Giovacchino Cambiagi Fiorentino, 1772).

61. Pouqueville F. *Voyage Dans La Grèce*. (Firmin Didot, Paris, 1820).

62. Psonis, N. *et al.* Identification of the 18 World War II executed citizens of Adele, Rethymnon, Crete using an ancient DNA approach and low coverage genomes. *Forensic Sci Int Genet* **71**, 103060 (2024).

63. Katsikaros, N. *The Vendetta in Mani.* (Self Published, Athens, 1933).

64. Cyriac of Ancona. *Later Travels.* (Harvard University Press, Cambridge, Massachusetts, 2003).

65. Taifakos, I. *Unpublished Documents of the Felouris Family.* (1968).

66. Y-chromosome DNA haplotree: Family Tree DNA Ltd. https://www.familytreedna.com/public/y-dna-haplotree (2025).

67. QGIS. QGIS v.3.12. https://qgis.org/ (2024).

68. ESRI. ESRI Satellite (ArcGIS/World_Imagery). https://server.arcgisonline.com/ArcGIS/rest/services/World_Imagery/MapServer/tile/%7Bz%7D/%7By%7D/%7Bx%7D (2024).

69. ESRI. World Terrain Base Map. https://server.arcgisonline.com/ArcGIS/rest/services/World_Terrain_Base/MapServer/tile/%7Bz%7D/%7By%7D/%7Bx%7D (2024).

70. Seifried, R. M. The Stone-Built Palaiomaniatika of the Mani Peninsula, Greece. in *Deserted villages : perspectives from the eastern Mediterranean* (eds. Seifried, R. M. & Brown Stewart, D. E.) 153–205 (The Digital Press at the University of North Dakota, 2021).

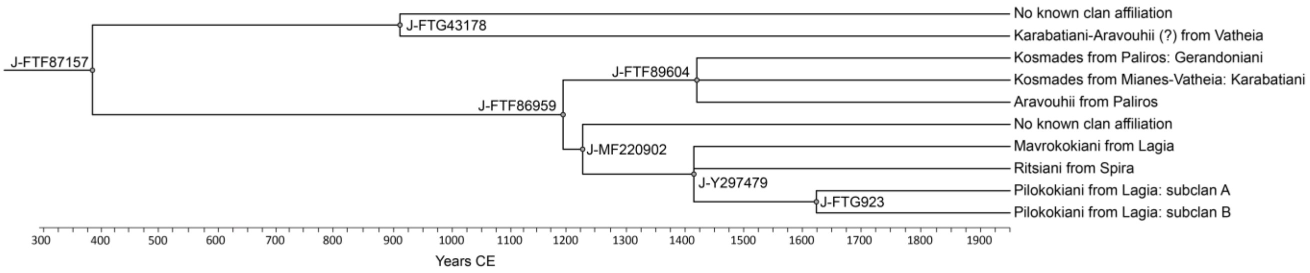

Figure S1. Phylogeny of haplogroup J-FTF87157, as reconstructed by FamilyTreeDNA's haplotree.

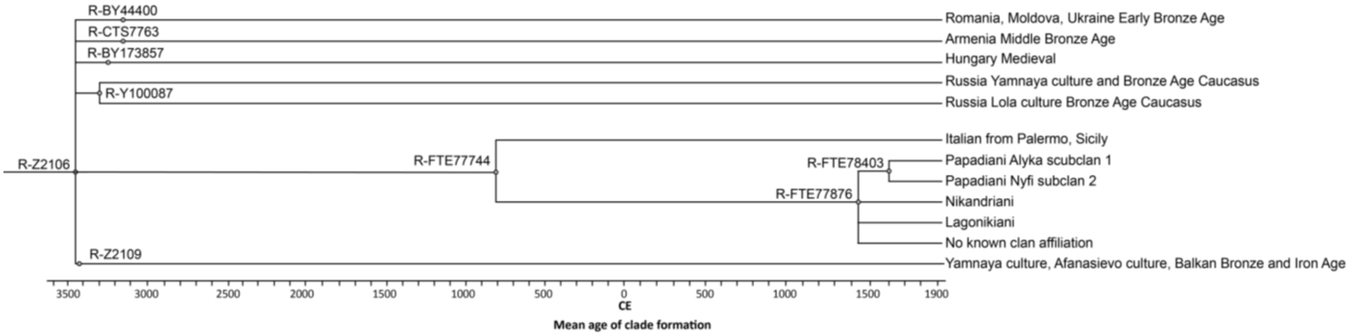

Figure S2. Phylogeny of haplogroup R-Z2106, as reconstructed by FamilyTreeDNA's haplotree.

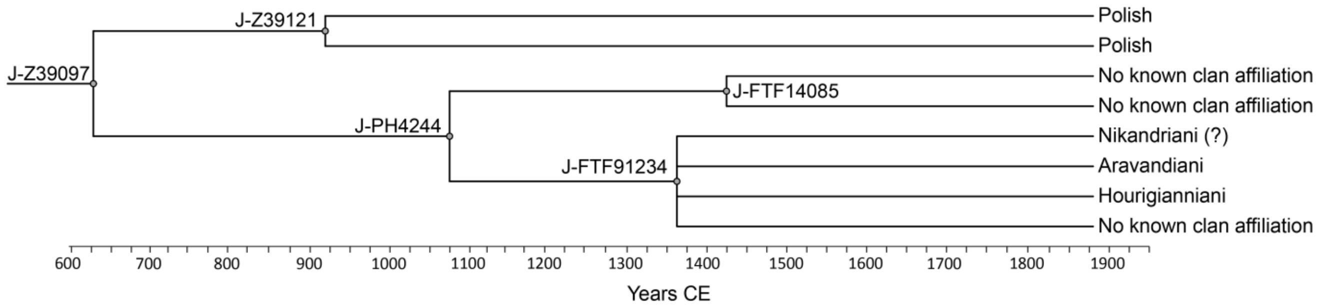

Figure S3. Phylogeny of haplogroup J-Z39097, as reconstructed by FamilyTreeDNA's haplotree.

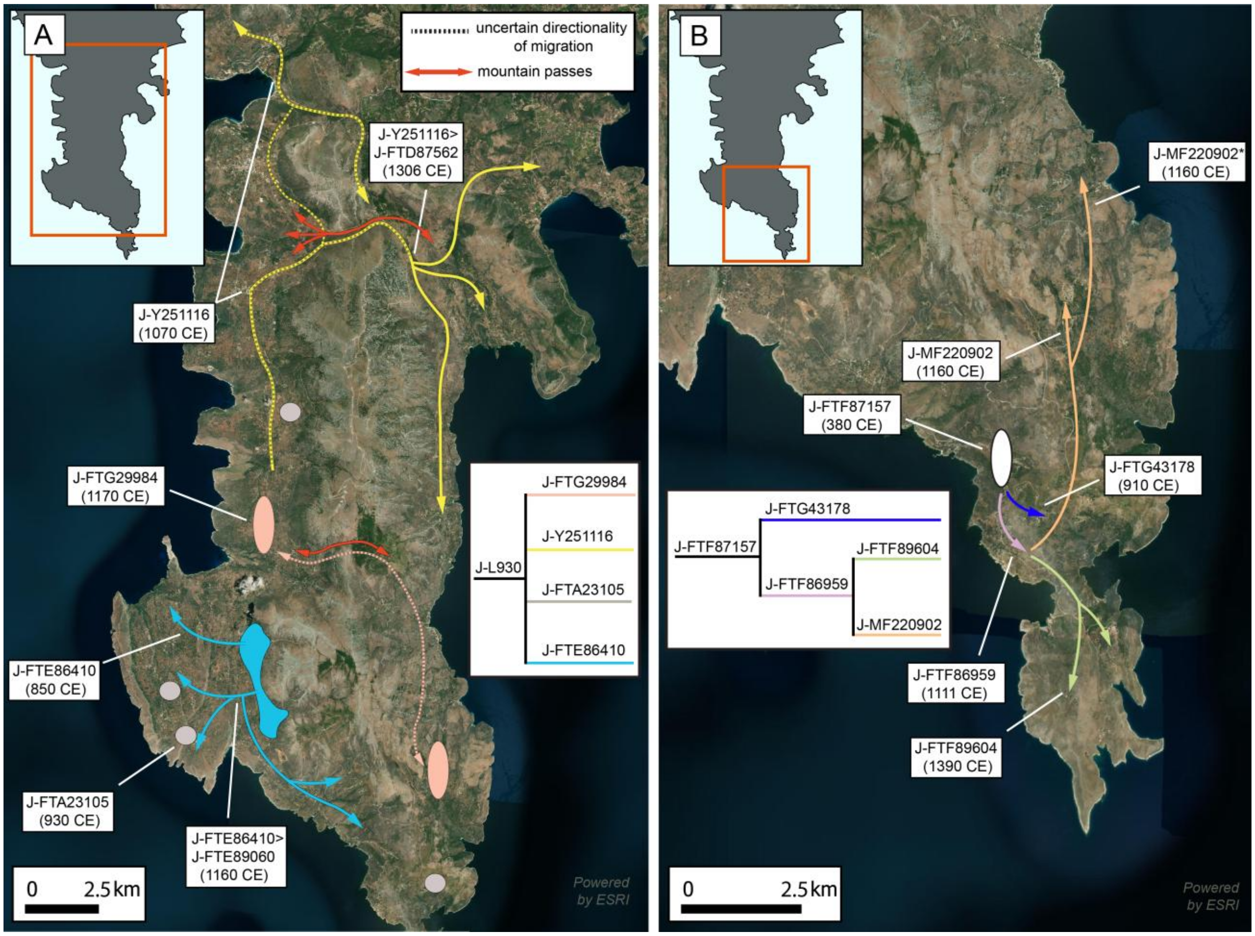

Figure S4. Hypothetical reconstruction of settlement patterns in Deep Mani, according to the branching patterns of haplogroups J-L930 and J-FTF87157 – the predominant paternal lineages among Deep Maniots. Dashed arrows indicate that the geographical origin of a particular migrational wave cannot be determined. A) Settlement patterns of J-L930-carrying individuals. Note that subclade J-FTA23105 is represented by circles, highlighting and its disjunct distribution. The location of mountain passes and road networks over the last 600 years were obtained from the work of Saitas[24]. B) Settlement patterns of descendants carrying haplogroup J-FTF87157. The satellite image of the Mani Peninsula was created with QGIS 3.40.0.48[67] using a basemap from the Esri World Terrain Base Map, ArcGIS Online (Sources: Esri, USGS, NOAA)[68,69]. Inset maps of Deep Mani indicate the location that is shown in higher magnification in each panel. The exact location of the Deep Mani peninsula within Greece can be found in Fig. 1 of the main text.

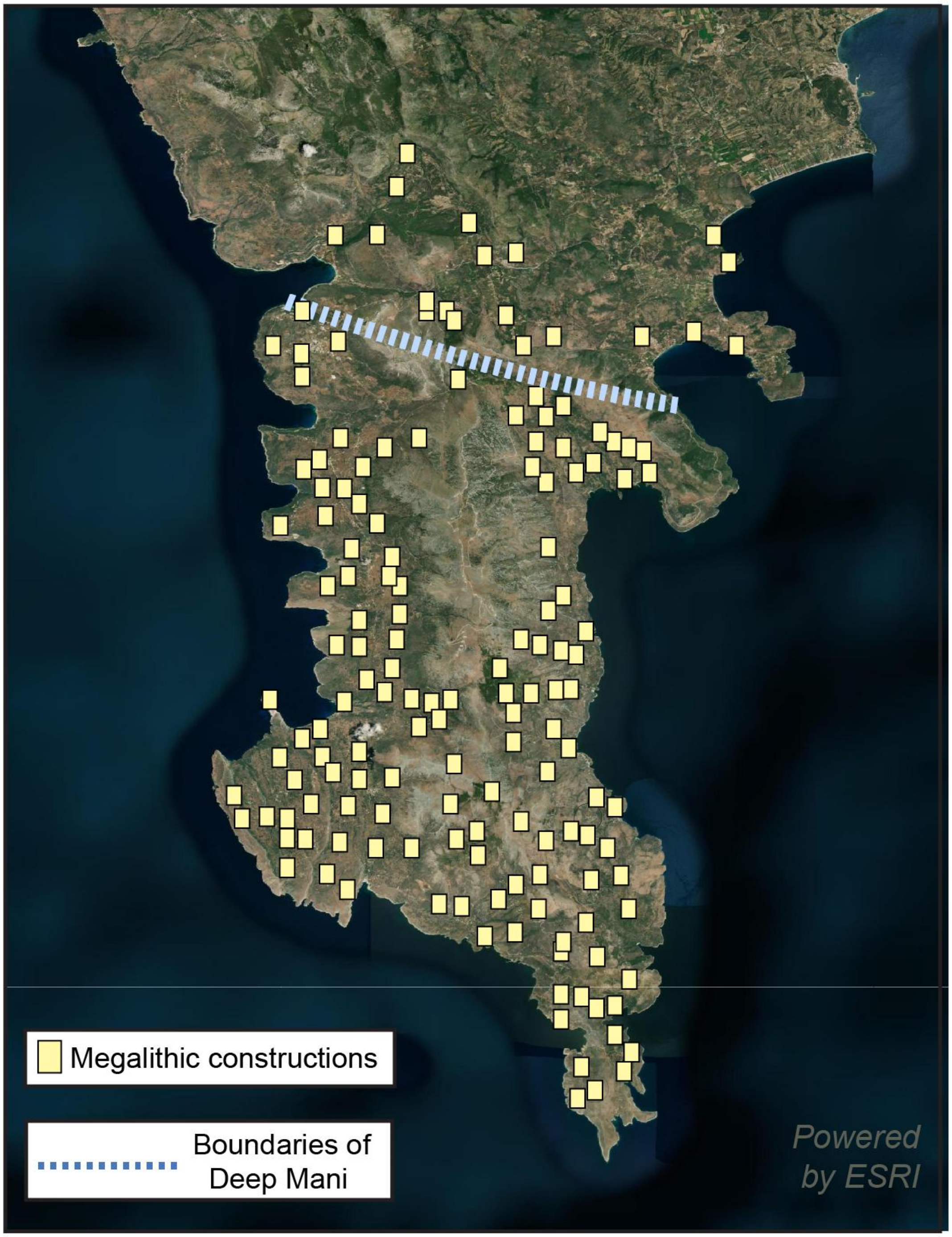

Figure S5. Map of megalithic constructions on the Mani Peninsula, based on the studies of Saitas[24,35] and Seifried[70]. Note that only a handful of megalithic constructions extend beyond the northern borders of Deep Mani, in the transitional zone of Outer Mani. The satellite image of the Mani Peninsula was created with QGIS 3.40.0.48[67] using a basemap from the Esri World Terrain Base Map, ArcGIS Online (Sources: Esri, USGS, NOAA)[68,69].

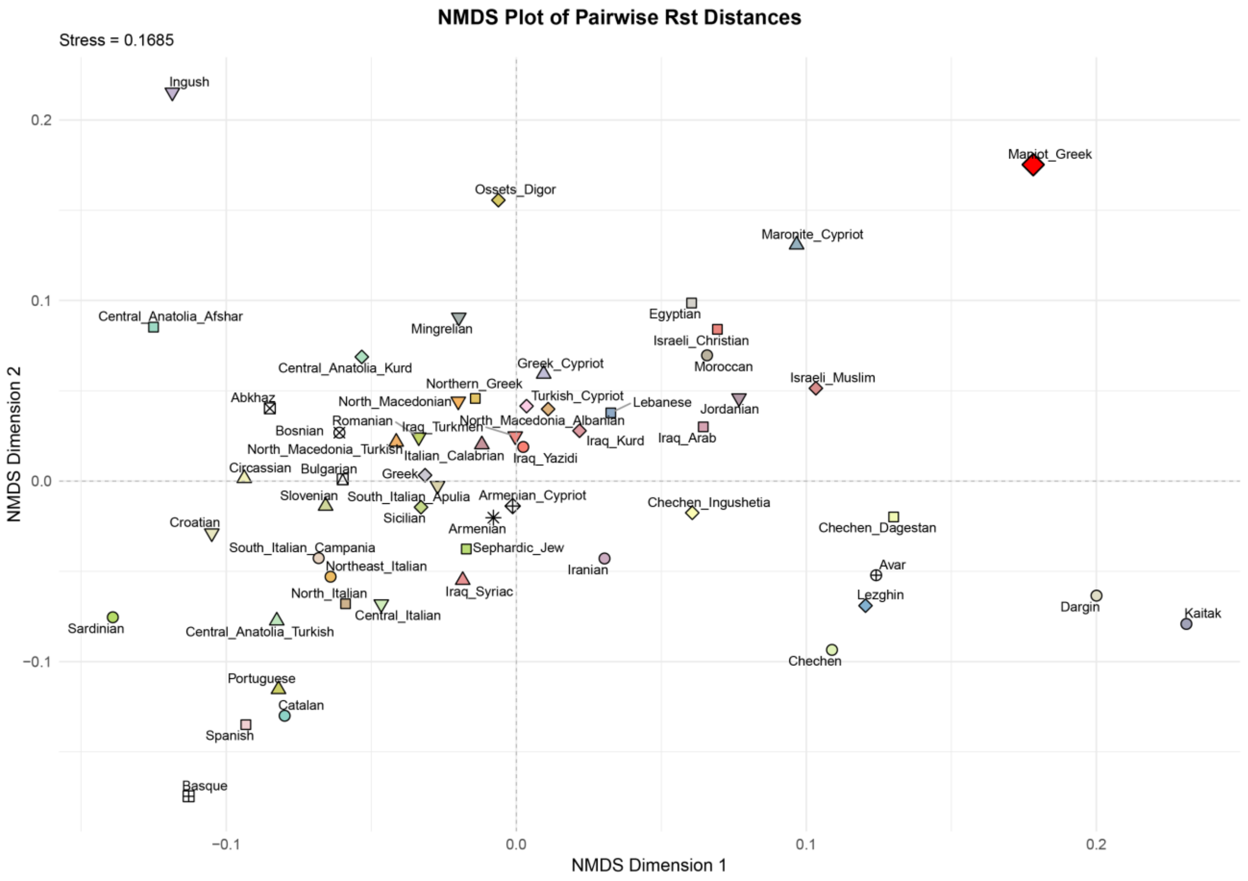

Figure S6. NMDS plot based on pairwise RST distances.

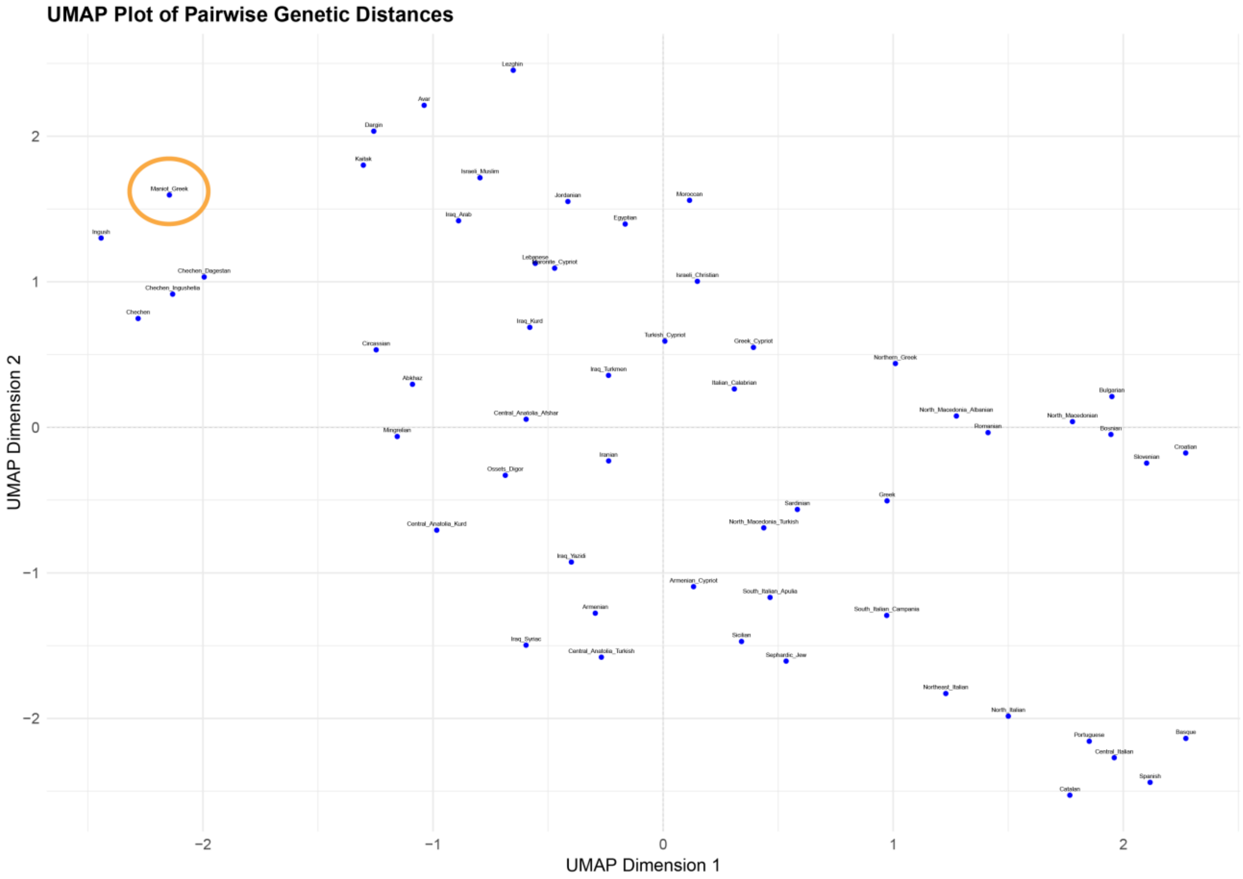

Figure S7. UMAP plot based on pairwise Rogers' distances.
