# Supplementary material for: Uniparental analysis of Deep Maniot Greeks reveals genetic continuity from the pre-Medieval era
Source: Commun Biol. 2026 Feb 4;9:157. doi: 10.1038/s42003-026-09597-9 (PMC12873217; doi:10.1038/s42003-026-09597-9)
Supplement: Supplementary file 2 — Description of additional supplementary files. [file 42003_2026_9597_MOESM2_ESM.pdf]

Description of Additional Supplementary files

File name: Supplementary Data 1
Description: Deep Maniot uniparental data

File name: Supplementary Data 2
Description: Mainland Greek and Deep Maniot Greek Y-DNA percentages

File name: Supplementary Data 3
Description: Greece uniparentals

File name: Supplementary Data 4
Description: West Eurasian 17 Y-STR list

File name: Supplementary Data 5
Description: Roger's-RST distances

File name: Supplementary Data 6
Description: Maniot Y-17 STR matches

File name: Supplementary Data 7
Description: IBD-sharing J-L930

File name: Supplementary Data 8
Description: qpAdm models

File name: Supplementary Data 9

Description: E-V13 subclades Greece

File name: Supplementary Data 10

Description: Deep Maniot Y-DNA TMRCA's

File name: Supplementary Data 11

Description: Deep Maniot clan TMRCA's

File name: Supplementary Data 12

Description: Clan origin myths

File name: Supplementary Data 13

Description: Outer Maniot Y-DNA

File name: Supplementary Data 14

Description: mtDNA details

File name: Supplementary Data 15

Description: mtDNA network analysis
